# Tumor Extracellular Vesicles Aggravate Mitochondrial Damage in Myocardial Ischemia/Reperfusion Injury

**DOI:** 10.1002/advs.202417493

**Published:** 2025-08-11

**Authors:** Zhongting Mei, Qian Liu, Guoxin Liu, Manjie Zhang, Jiaxin Fang, Xuting He, Xueqi He, Zhengwei Qin, Ronghua Liu, Chuang Liu, Hongyu Ji, Yuechao Dong, Ye Yuan, Baofeng Yang, Weijie Du

**Affiliations:** ^1^ State Key Laboratory of Frigid Zone Cardiovascular Diseases (SKLFZCD) Department of Pharmacology (State Key Laboratoray‐Province Key Laboratories of Biomedicine‐Pharmaceutics of China Key Laboratory of Cardiovascular Research, Ministry of Education) College of Pharmacy Harbin Medical University Harbin 150081 China; ^2^ Department of Pharmacy at the Second Affliated Hospital State Key Laboratory of Frigid Zone Cardiovascular Diseases (SKLFZCD) Harbin medical university Harbin 150081 China; ^3^ College of Pharmacy Harbin Medical University Harbin 150081 China

**Keywords:** cancer, extracellular vesicles, miR‐485‐3p, mitochondrial dysfunction, myocardial ischaemia/reperfusion, percutaneous coronary intervention, PPARGC1A/PGC‐1α

## Abstract

Cancer patients have a higher risk of all types of cardiovascular diseases (CVDs). Cardiologists are encountering a growing number of cancer patients with CVDs, and an increasing number of cancer patients undergoing percutaneous coronary intervention (PCI). The mechanistic link between cancer and CVDs remains elusive. Here, the meta‐analysis shows the increased incidence ratio of all‐cause mortality and cardiovascular (CV) mortality in patients undergoing PCI with cancer compared with non‐cancer. We experimentally demonstrated that cancer aggravated mitochondrial dysfunction and myocardial ischemia/reperfusion (I/R) injury in two models of lung cancer in mice. Plasma extracellular vesicles (EVs) derived from cancer mice exacerbated cardiac I/R injury in vivo and in vitro, while inhibition of tumor EVs secretion by lipid‐coated polyacrylic acid/calcium phosphate nanoparticles delivered a neutral sphingomyelinase inhibitor (GW4869) showed the opposite results. Lung‐specific miR‐485‐3p sponges mediated by Adeno‐associated virus 6 suppress cardiac damage and mitochondrial dysfunction in CC10‐KRAS^G12D^ mice post‐I/R. Mechanistically, PPARGC1A/PGC‐1α is post‐transcriptionally repressed by miR‐485‐3p in cardiomyocytes, leading to the inhibition of mitochondrial complex I subunits and ATP synthesis. Taken together, our findings reveal for the first time that cancer can aggravate cardiac injury and mitochondrial dysfunction by releasing miR‐485‐3p‐enriched extracellular vesicles derived from cancer cells.

## Graphical abstract

This study reveals a novel mechanistic connection between cancer and ischemia/reperfusion (I/R)‐induced cardiac injury. The meta‐analysis demonstrates significantly higher incidence ratios of both all‐cause mortality and cardiovascular mortality in cancer patients undergoing percutaneous coronary intervention (PCI) compared to non‐cancer patients. The findings suggest that cancer exacerbates cardiac injury and mitochondrial dysfunction through the release of miR‐485‐3p‐enriched extracellular vesicles originating from lung cancer cells. The graphical abstract is created using Scidraw and BioRender.

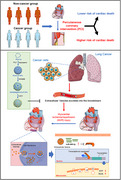

## 1 Introduction

Cancer and cardiovascular diseases (CVDs) represent two of the most life‐threatening diseases worldwide, exhibiting overlapping risk factors and shared pathogenic mechanisms^[^ ^1^ ^]^ Several studies have shown that cardiac remodeling and heart failure induced by myocardial infarction or transverse aortic constriction/pressure overload promote tumor growth.^[^ ^2^, ^3^, ^4^ ^]^ There are a number of evidence supporting that chemotherapy and radiotherapy could result in cardiotoxicity.^[^ ^5^ ^]^ However, the occurrence of cardiac wasting in several cancers before starting any anti‐cancer therapy is reported in the clinic.^[^ ^6^, ^7^ ^]^ Notably, recent clinical evidence has reported the prevalence of CVDs in patients with malignancies and demonstrated patients with lung cancer exhibit the highest CVDs burden (43%) across all malignancy types.^[^ ^8^ ^]^ Active cancer or a history of cancer is increasingly prevalent among coronary artery disease patients undergoing percutaneous coronary intervention in real‐world clinical settings. A retrospective analysis revealed a significantly higher incidence rate ratio of all‐cause mortality in patients with cancer compared to those without cancer following percutaneous coronary intervention,^[^ ^9^ ^]^ its effect on cardiovascular (CV) mortality remains controversial. These studies enhance our understanding of the cause‐and‐effect relationship between these two diseases.

Mitochondria serve as the primary energy factories in cells, generating ATP through oxidative phosphorylation. Mitochondria are exceptionally abundant in cardiomyocytes, constituting ≈30% of cell volume, and are strategically positioned between myofibrils and beneath the sarcolemma to efficiently supply ATP for cardiac contraction.^[^ ^10^, ^11^ ^]^ Mitochondrial homeostasis is crucial for maintaining cardiac function.^[^ ^12^ ^]^ In various CVDs, mitochondrial dysfunction plays a central pathogenic role.^[^ ^13^, ^14^ ^]^ Given their central role in disease pathogenesis, targeting the mitochondrial dysfunction axis‐particularly the maladaptive interplay between electron transport chain (ETC) impairment, ROS overproduction, and mitochondrial membrane potential (ΔΨm) collapse represents a promising therapeutic strategy, with strategies focusing on enhancing mitochondrial quality control, reducing oxidative stress, and improving bioenergetics.^[^ ^13^, ^15^, ^16^ ^]^

Extracellular vesicles (EVs) are a heterogeneous group of cell‐derived membranous structures comprising exosomes and microvesicles, which originate from the endosomal system or which are shed from the plasma membrane, respectively.^[^ ^12^, ^17^ ^]^ They are secreted into the extracellular environment by most cell types, where they can be internalized by recipient cells through various uptake mechanisms.^[^ ^18^ ^]^ Extracellular vesicles carry diverse molecular cargoes, including proteins, nucleic acids (DNA and RNA), which play a pivotal role in intercellular communication. These EVs‐derived biomolecules can change the fate of recipient cells by autocrine and paracrine mechanisms. It has been reported that extracellular vesicles from cancer cells effectively remodel distant microenvironments to enhance metastasis in multiple cancer types.^[^ ^19^ ^]^ Extracellular miRNAs are recognized as important mediators of intercellular communications and potential candidates for therapy of disease.^[^ ^20^ ^]^ We have previously uncovered that myocardial infarction (MI) suppresses erastin‐induced ferroptosis by releasing miR‐22‐3p‐enriched exosomes derived from cardiomyocytes.^[^ ^21^ ^]^ However, whether the cancer cells secreted EVs can modulate myocardial ischemia/reperfusion (I/R) injury remains to be yet studied.

We proposed, based on the findings reported by above‐mentioned studies, that cancer aggravates I/R through a remote signal communication mediated by tumor‐derived extracellular vesicles and executed by miRNAs carried by the extracellular vesicles. The goal of our study was to examine our hypotheses with three specific objectives: to explore the effects of lung cancer on I/R injury, to examine if lung tumor‐secreted EVs mediate the connection between tumor and I/R, and to decipher the signaling mechanisms underlying the tumor‐heart interactions in the setting of I/R. Our experimental results indicate that EVs secreted from lung cancer cells play an essential role in aggravating mitochondrial damage thereby promoting I/R injury. MiR‐485‐3p is packaged into extracellular vesicles and transferred from tumor cells to cardiomyocytes, where it downregulates the expression of peroxisome proliferator‐activated receptor gamma coactivator‐1 alpha (PGC‐1α), a critical regulator of the mitochondria and energy metabolism homeostasis.

## 2 Results

### 2.1 Lung Cancer Aggravates Cardiac I/R Injury

**Figure 1:**
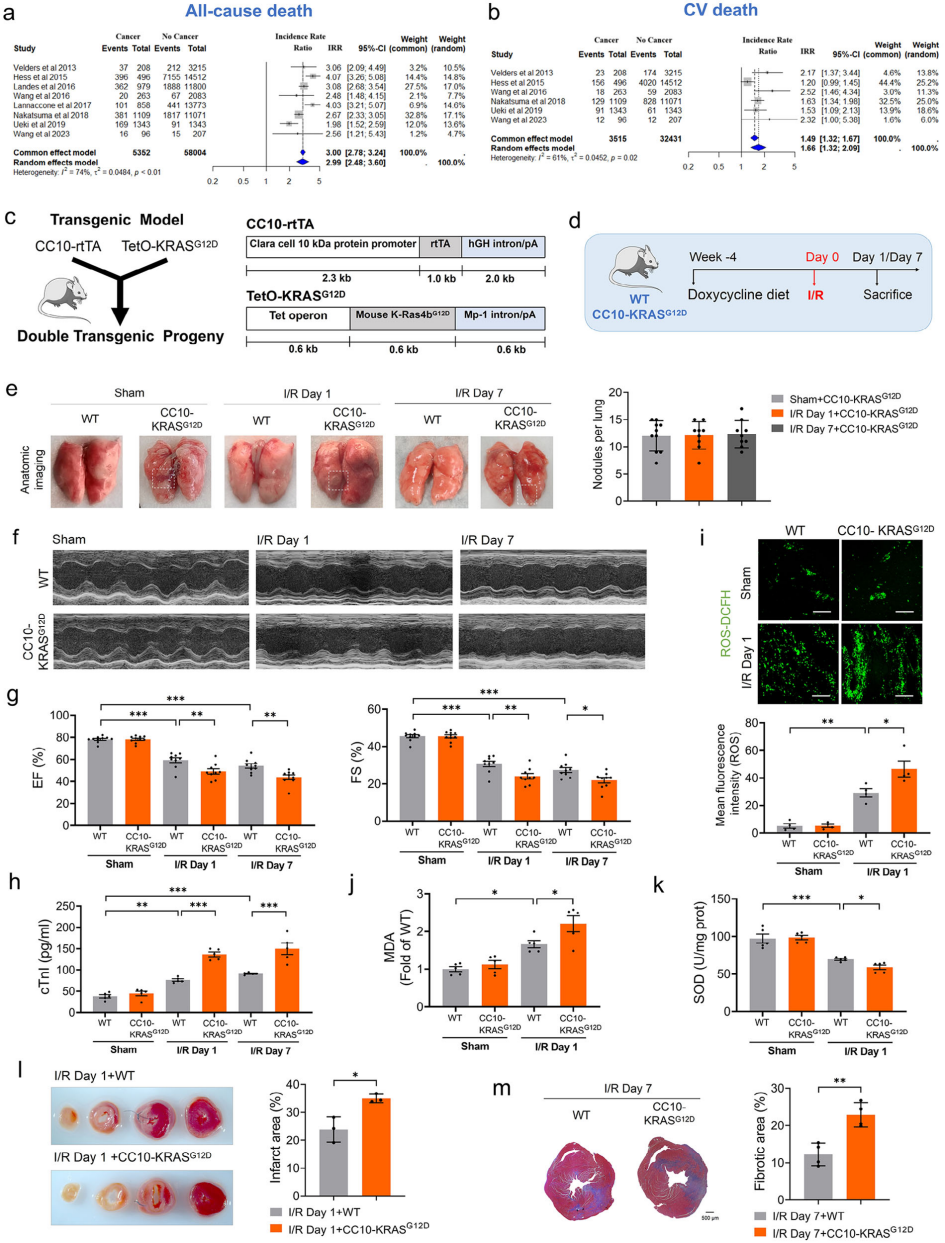
Lung cancer aggravates cardiac I/R injury a,b) Incidence rate ratio of all‐cause and cardiovascular mortality in patients with versus without cancer undergoing percutaneous coronary intervention; c) Generation of plasmid constructs and bitransgenic  mice; d) Schematic presentation on the time‐line of in vivo animal model construction experiment, WT and CC10‐KRAS^G12D^ mice were treated with ≈0.1 mg kg^−1^ doxycycline daily for 4 weeks, the mice were subjected to I/R or sham operation for 1 day or 7 days, respectively; e) Representative images of anatomic imaging of lung and lung tumor statistical data in mice (N = 9‐10/group); f‐g) Representative images of echocardiographs and statistical data on EF%, FS% (N = 9‐10/group); h) Plasma cTnI levels were detected by ELISA assay (N = 5/group); i) Representative images and quantitative results of ROS (Bar: 40 µm) (N = 4/group); j,k) The levels of MDA and SOD were measured by ELISA assay (N = 5/group); l) Infarct area of I/R mice by TTC staining (N = 3/group); m) Representative images of Masson's trichrome‐stained transverse mid slices of LVs 7 days post‐IR and quantification of percent LV fibrosis (Bar: 500 µm) (N = 4/group). Data are expressed as mean ± SEM. ^*^ *p* < 0.05; ^**^ *p* < 0.01; ^***^ *p* < 0.001.

Despite the increasing number of cancer patients undergoing PCI, limited data are available on the incidence rate ratios (IRR) of all‐cause and CV mortality in this population, with existing studies reporting inconsistent findings. Therefore, we sought to compare all‐cause mortality and CV mortality outcomes between patients undergoing PCI with versus without a cancer diagnosis (Table S1, Supporting Information). Overall, eight observational studies with available data were included to calculate the IRR for all‐cause and CV mortality (Figure S1, Supporting Information), encompassing ≈63 000 individuals (Table S2, Supporting Information).^[^ ^1^, ^22^, ^23^, ^24^, ^25^, ^26^, ^27^, ^28^ ^]^ Pairwise meta‐analysis demonstrated that cancer patients undergoing PCI had significantly higher rates of both all‐cause mortality (IRR 2.99, 95% CI 2.48 – 3.60, P < 0.001) and CV mortality (IRR 1.66, 95% CI 1.32 – 2.09, P = 0.001) (**Figure** **1**a,b). Risk of bias assessment using the Newcastle‐Ottawa Scale indicated low bias across domains (Table S3, Supporting Information). Heterogeneity was observed (P < 0.05), necessitating the use of a random effects model. Although funnel plots showed slight asymmetry, no significant publication bias was detected (Figures S2 and S3, Supporting Information).

To investigate the effects of the tumor on myocardial ischemia/reperfusion (I/R) injury, we employed a conditional transgenic system wherein lung‐specific tumorigenesis was induced in bitransgenic Tet‐On CC10‐rtTA/(tetO)7‐KRAS^G12D^ (CC10‐KRAS^G12D^) mice through dietary administration of doxycycline. Both wild‐type (WT) and bitransgenic mice were administered doxycycline at a dose of ≈0.1 mg kg^−1^ per day via dietary supplementation for 4 weeks prior to sham or I/R surgery (Figure S4a, Supporting Information). We performed a time series analysis of the CC10‐KRAS^G12D^ mouse heart after 45 min of ischemia followed by reperfusion (24 h or 7 days; Figure 1c,d). No significant differences in lung total tumor burden were observed across experimental groups in the CC10‐KRAS^G12D^‐driven lung cancer mouse model (p > 0.05), thereby ruling out tumor burden as a confounding variable in the study (Figure 1e). H&E staining of major organs (heart, spleen, kidney, brain, liver) revealed no significant histopathological alterations between WT and CC10‐KRAS^G12D^‐driven lung cancer mice, with comparable tissue architecture observed in both groups (Figure S4b, Supporting Information). Echocardiographic assessments at baseline indicated preserved cardiac function in tumor‐bearing mice post‐sham surgery, with left ventricular ejection fraction (EF%) and fractional shortening (FS%) values equivalent to WT controls. However, lung cancer‐bearing mice exhibited the aggravated cardiac dysfunction compared with control mice, as demonstrated by reduced EF% and FS% at day 1 and 7 post‐I/R (Figure 1f,g). In parallel with the increase in LVIDs post‐I/R, LVIDd showed no significant change (Figure S4d, Supporting Information). No significant alterations in heart weight‐to‐body weight ratio were observed in lung cancer‐bearing mice as compared to the WT non‐cancer mice following sham or I/R operation (Figure S4e,f, Supporting Information). We next measured the plasma cardiac troponin I (cTnI) levels to investigate the tumor‐mediated potentiation on cardiac injury. As shown in Figure 1h, I/R on day 1 increased plasma cTnI levels in WT non‐cancer mice, with a much more pronounced elevation observed in lung cancer‐bearing counterparts. A similar trend was observed after extending the time to 7 days. Additionally, lung tumor‐bearing mice exhibited a marked elevation in oxidative stress compared to non‐tumor controls 1 day post‐I/R, as manifested by increased cardiac tissue of reactive oxygen species (ROS) and malondialdehyde (MDA) levels, and decreased speroxide dismutase (SOD) activity (Figure 1i–k). Moreover, a larger infarct size was observed in cancer‐bearing mice than in non‐cancer controls at day 1 after I/R (Figure 1l). Masson's trichrome staining analysis revealed markedly increased cardiac fibrosis area in lung cancer‐bearing mice compared with control mice at day 7 after I/R, whereas no significant fibrosis was observed in the heart of lung cancer‐bearing mice and non‐cancer controls after sham operation (Figure 1m; Figure S4c, Supporting Information).

To further corroborate these findings, an orthotopic transplantation of mouse Lewis lung carcinoma was established prior to I/R surgery (Figure S5a, Supporting Information). Tumor development was confirmed (Figure S5b, Supporting Information). Consistently, lung cancer‐bearing mice subjected to I/R (24 h) showed more severe cardiac damage and worse cardiac function than non‐cancer mice after I/R (Figure S5c–e, Supporting Information). We next examined the plasma lactate dehydrogenase (LDH) and cTnI levels to investigate the role of tumor on cardiac injury. As anticipated, lung cancer‐bearing mice exhibited higher levels of plasma LDH and cTnI than those in non‐cancer controls after I/R (Figure S5f,g, Supporting Information). Parallelly, lung tumor‐bearing mice subjected to I/R exhibited significantly higher ROS production and MDA levels, along with lower SOD levels, compared to non‐tumor mice after I/R (Figure S5h–j, Supporting Information). HW/BW ratio and body weight were unchanged in lung cancer‐bearing mice compared to non‐cancer mice (Figure S5k,l, Supporting Information). These results suggest that lung cancer aggravates cardiac I/R injury in vivo.

### 2.2 Extracellular Vesicles from the Mice Bearing Lung Tumor Augments Cardiac I/R Injury

**Figure 2:**
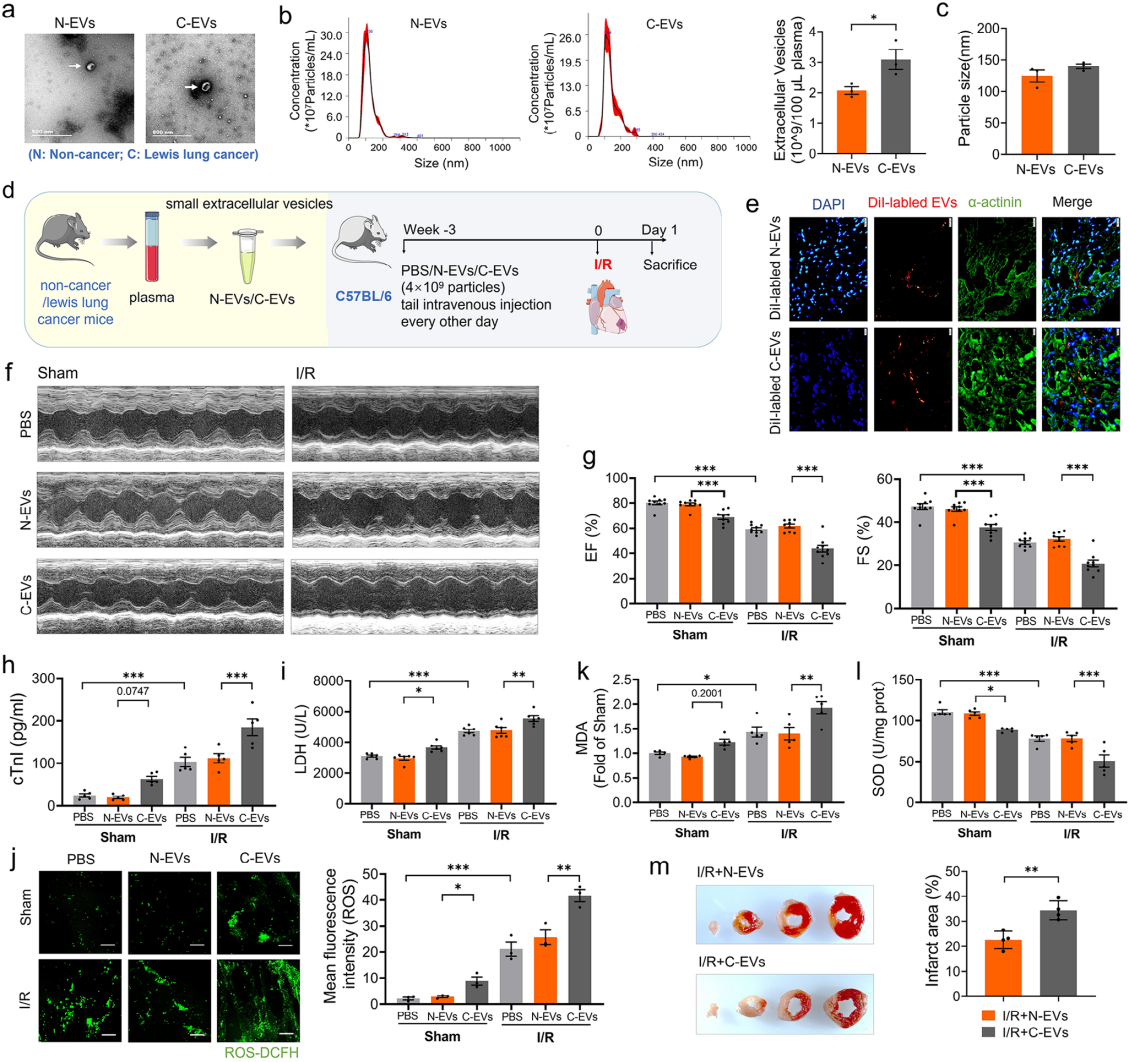
Extracellular vesicles from the mice bearing lung tumor augments cardiac I/R injury a) Extracellular vesicles morphology was characterized by TEM in isolated from mouse plasma (N‐EVs: Non‐cancer‐EVs, C‐EVs: Lung cancer‐EVs); b,c) Particle size distribution was determined by nanosight tracking analysis (N = 3/group); d) Schematic presentation on the time‐line of in vivo EVs tail intravenous injection experiment, mice were inoculated with an equal number of N‐EVs or C‐EVs (4 × 10^9^ particles) every other day prior to the establishment of either Sham or I/R mouse models; e) Immunofluorescence imaging confirmed that EVs were retained within the heart by tail intravenous injection of DiI‐labelled N‐EVs /C‐EVs for 48 h (Bar: 20 µm); f,g) Representative images of echocardiographs and statistical data on EF%, FS% (N = 9/group); h,i) Plasma LDH and cTnI levels were detected by ELISA assay (N = 5‐6/group); j) Representative images and quantitative results of ROS using ROS fluorescence staining (Bar: 40 µm) (N = 3/group); k,l) The levels of MDA and SOD were measured by ELISA assay (N = 5/group); m) Infarct area of I/R mice by TTC staining (N = 4/group). Data are expressed as mean ± SEM. ^*^ *p* < 0.05; ^**^ *p* < 0.01; ^***^ *p* < 0.001.

The observations presented above prompted us to investigate the mechanisms underlying the detrimental effects of lung cancer on cardiac I/R injury. Extracellular vesicles containing various constituents (protein, DNA, RNA etc.) secreted by cells are taken up by distant cells, where they may affect cellular function and behavior.^[^ ^29^ ^]^ To determine whether plasma EVs from lung cancer‐bearing mice could exacerbate I/R injury, we isolated plasma EVs from cancer‐bearing and non‐cancer mice by ultra‐centrifugation and examined them using electron microscopy (**Figure** **2**a). The Nanoparticle tracking analysis (NTA) was performed to characterize EVs morphology and size distribution. The analysis revealed a particle concentration of 2.07 × 10^10^ particles mL^−1^ for EVs from non‐cancer mice (N‐EVs) and 3.09 × 10^10^ particles mL^−1^ for cancer‐derived EVs (C‐EVs) (Figure 2b). No significant difference was observed in the average diameter of spherical vesicles between N‐EVs (124.5 nm) and C‐EVs (139.8 nm), both falling within the typical size range for extracellular vesicles (Figure 2c). To investigate the effects of plasma EVs on cardiac I/R injury, mice was received intravenous injections of either N‐EVs or C‐EVs (4 × 10⁹ particles) via the tail vein every other day prior to I/R surgery.^[^ ^30^ ^]^ Control animals received equal volumes of PBS following the same regimen. Mice were subjected to cardiac I/R surgery following tail vein injection of PBS, N‐EVs and C‐EVs (Figure 2d). We next performed immunofluorescence staining to determine whether EVs (4 × 10^9^ particles) could be utilized by cardiac tissue, and the result showed that EVs can still retain within the heart at 48 h after tail intravenous injection (Figure 2e). Echocardiography analyses demonstrated that C‐EVs treatment exacerbated cardiac dysfunction induced by I/R compared with N‐EVs‐treated mice. Intriguingly, in sham‐operated mice, C‐EVs administration alone induced marked cardiac dysfunction compared to both N‐EVs and PBS‐treated controls (Figure 2f,g). We then observed that C‐EVs treatment aggravated cardiac injury as reflected by increased plasma levels of cTnI and LDH as compared to the N‐EVs‐treated mice of either sham or I/R operation (Figure 2h,i). Also, we observed a remarkable increase in ROS production and MDA level, and a striking reduction in SOD level in C‐EVs‐treated mice compared to N‐EVs treatment with and without I/R (Figure 2j–l). Parallelly, the infarct size was larger in I/R mice receiving C‐EVs compared to those receiving N‐EVs (Figure 2m). In addition, Immunohistochemical staining revealed no statistically significant differences in the expression of either CD68 or Ly‐6G between the C‐EVs‐treated mice and the N‐EVs‐treated mice, suggesting that tumor‐derived EVs may exacerbate cardiac injury through mechanisms independent of cardiac‐resident immune cells in I/R model (Figure S6, Supporting Information). Collectively, the above obtained results indicate that EVs derived from mice bearing lung tumor exacerbate cardiac injury post I/R.

### 2.3 Inhibition of Tumor EVs Secretion Suppress Cardiac Injury in I/R Mouse Model

**Figure 3:**
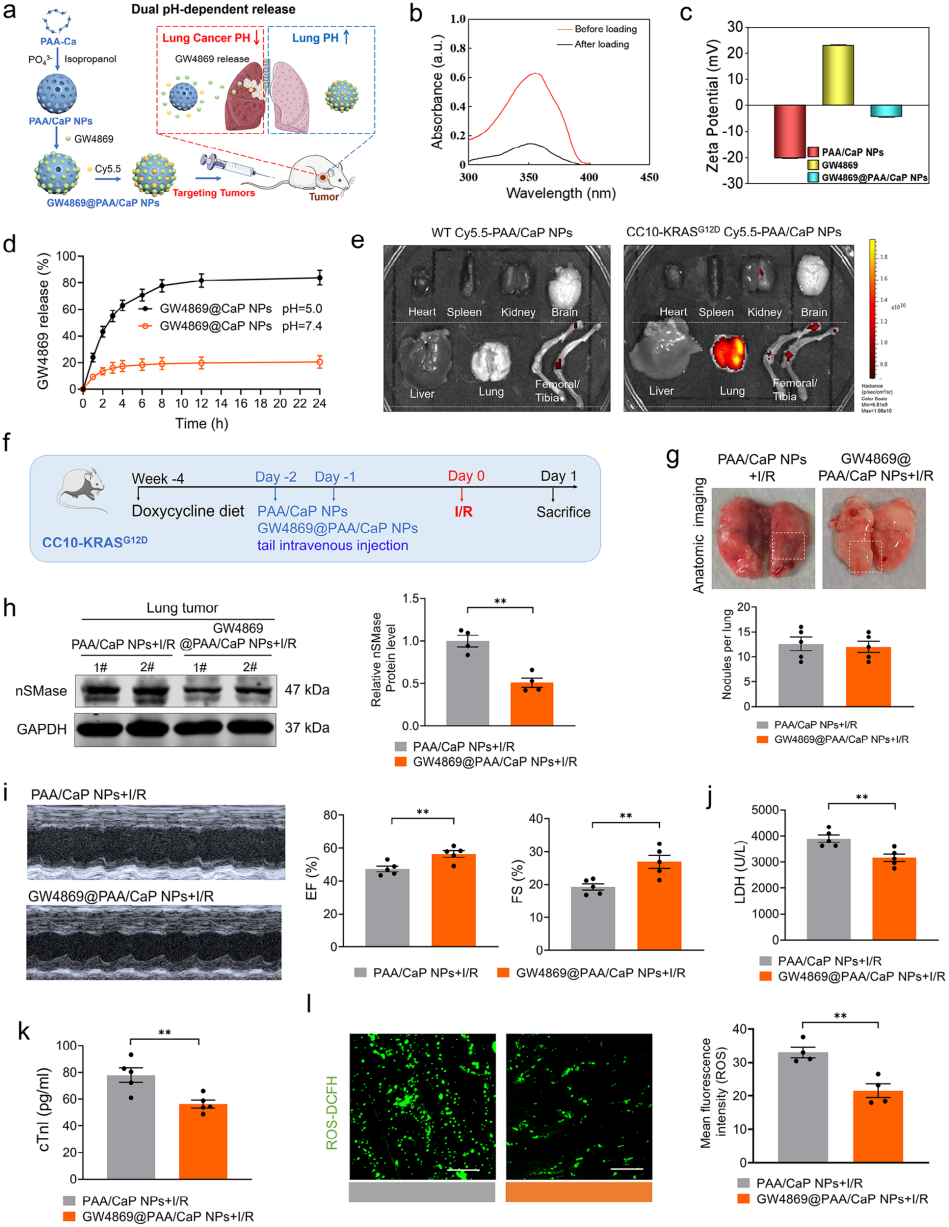
Inhibition of tumor EVs secretion suppress cardiac injury in I/R mouse model a) Schematic illustration of the synthetic strategy for the GW4869@PAA/CaP NPs as dual pH‐responsive drug carriers for targeting tumor therapy in vivo; b) UV‐vis absorption spectra of GW4869 solutions before (red curve) and after (black curve) loading with PAA/CaP NPs; c) The zeta potential of PAA/CaP NPs, GW4869 and GW4869@PAA/CaP NPs; d) The release experiment of GW4869@PAA/CaP NPs in vitro; e) GW4869@PAA/CaP NPs were intravenously injected into the mice (WT and CC10‐KRAS^G12D^). Respective bioluminescence images of heart, spleen, kidney, brain, liver, lung and femoral/tibia in 6 h; f) Schematic presentation on the time‐line of in vivo targeted inhibition of extracellular vesicles secretion experiment, GW4869@PAA/CaP NPs (GW4869 1 mg kg^−1^) were administered to CC10‐KRAS^G12D^ mice twice via the tail intravenous injection with an interval of 24 h. The same amounts of PAA/CaP NPs were treated as control. After 24 h, the mice were subjected to I/R operation for 1 day; g) Representative anatomic images and statistics analysis of lung tumors (N = 5/group); h) Quantification and representative images for nSMase of lung tumors by western blot (N = 4/group); i) Representative images of echocardiographs and statistics analysis of EF% and FS% after different treatments (N = 5/group); j,k) Plasma LDH and cTnI levels were detected by ELISA assay (N = 5/group); l) Representative fluorescent images of the intracellular ROS in cultured cardiomyocytes after different treatments (N = 4/group, Bar: 40 µm). Data are expressed as mean ± SEM. ^**^ *p* < 0.01; ^***^ *p* < 0.001.

Next, we asked ourselves whether blocking EVs release from lung tumor‐bearing mice could alleviate cardiac I/R injury. To answer this question, we employed GW4869 – a neutral sphingomyelinase inhibitor known to inhibit EVs secretion and vesicle trafficking in vivo.^[^ ^31^ ^]^ Our previous reports have shown that lipid‐coated polyacylic acid/calcium phosphate nanoparticles (PAA/CaP NPs), a new dual pH‐responsive drug delivery system for targeted cancer chemotherapy,^[^ ^32^ ^]^ were used as a GW4869 delivery vehicles for cancer (**Figure** **3**a). PAA/CaP NPs showed a uniform spherical shape with an average diameter of 175 ± 10 nm as confirmed by TEM and scanning electron microscopy (SEM) (Figure S7a, Supporting Information). Elemental mapping analysis, X‐ray photoelectron spectroscopy (XPS), fourier transform infrared (FTIR) and energy dispersive X‐ray (EDX) spectroscopy were performed to characterize the surface chemical composition of the PAA/CaP NPs (Figure S7b–g, Supporting Information). The elemental mapping results demonstrated a homogeneous distribution of elements throughout the PAA/CaP NPs. Collectively, these analyses confirmed the successful synthesis of PAA/CaP NPs. UV‐Vis absorption spectra revealed a characteristic absorption peak of GW4869 at 350 nm. Compared with free GW4869 solution, the GW4869 was effectively loaded into the PAA/CaP NPs (Figure 3b). Additionally, the change in zeta potential further confirmed the successful loading of GW4869 onto the PAA/CaP NPs (Figure 3c). PAA/CaP NPs exhibit pH‐responsive behavior, maintaining stability at physiological pH while dissolving rapidly under acidic conditions, thereby facilitating drug release.^[^ ^32^ ^]^ As shown in Figure 3d, PAA/CaP NPs remain stable in neutral phosphate buffered saline (PBS), which is similar to the normal tissue environment (pH 7.4), whereas the cumulative drug release amount attains 83.92% at mildly acidic environment (pH 5.0), which is similar to the extracellular pH of tumors. To track nanoparticle distribution, the hydrophilic fluorescent dye Cy5.5 was conjugated to the PAA/CaP NPs. Fluorescence emission spectrum analysis revealed that Cy5.5 exhibited strong fluorescence intensity centered ≈700 nm, and it successfully labeled PAA/CaP NPs with high efficiency (Figure S7h, Supporting Information). WT and CC10‐KRAS^G12D^ mice were intravenously injected with PAA/CaP NPs‐Cy5.5. Chemiluminescence imaging at 6 h post‐injection revealed that PAA/CaP NPs‐Cy5.5 exhibited superior lung tumor targeting than other organs in CC10‐KRAS^G12D^ mice compared with WT mice (Figure 3e). To inhibit extracellular vesicles biogenesis, GW4869@PAA/CaP NPs (1 mg kg^−1^ GW4869) were administered via tail vein injection, with PAA/CaP NPs alone serving as the control. After 24 h, I/R model was established (Figure 3f). No significant difference in total tumor burden was observed between the two groups (Figure 3g). GW4869 is a noncompetitive inhibitor of neutral sphingomyelinase (nSMase), which catalyzes the cleavage of sphingomyelin to phosphorylcholine and ceramide. This process is essential for the formation and release of extracellular vesicles from cells. Inhibition of nSMase has been demonstrated to suppress the release of EVs from cells.^[^ ^33^ ^]^ The nSMase expression of lung tumor tissue was decreased by GW4869@PAA/CaP NPs as compared with PAA/CaP NPs under the I/R‐operated conditions in CC10‐KRAS^G12D^ mice, there was no significant change in the expression level of nSMase in other organs (Figure 3h; Figure S8, Supporting Information). Cardiac contractile dysfunction was restored in GW4869@PAA/CaP NPs treated mice under the I/R‐operated conditions (Figure 3i). Consistently, the tumor‐targeted inhibition of EVs secretion also abrogated I/R‐induced injury, as indicated by decreased LDH release (Figure 3j), cTnI levels (Figure 3k), and ROS accumulation (Figure 3l). Together, these results demonstrate that lung cancer‐derived EVs aggravate cardiac injury.

### 2.4 EVs Treatment Promotes Cardiomyocyte Death In Vitro

**Figure 4:**
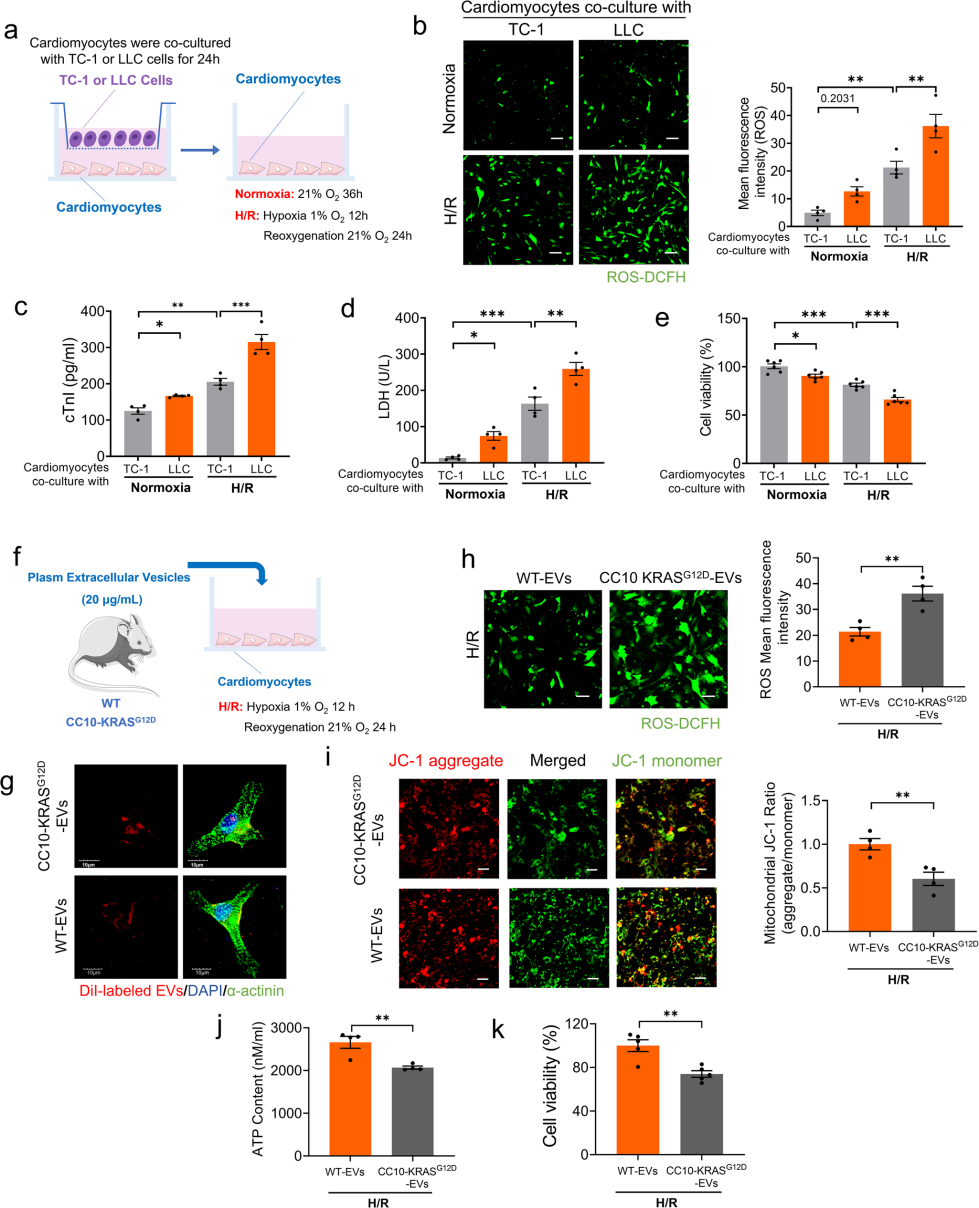
EVs treatment promotes cardiomyocyte death in vitro a) Schematic presentation on the time‐line of cells co‐cultured, after cardiomyocytes were co‐cultured with TC‐1 or LLC cells for 24 h, part of the cells were treated with hypoxia for 12 h and reoxygenation for 24 h; b) Representative images and quantitative results of ROS levels detected by ROS fluorescence staining (N = 4/group, Bar: 40 µm); c,d) Plasma LDH and cTnI levels were detected by ELISA assay (N = 4/group); e) Cardiomyocyte viability was detected by CCK‐8 assay (N = 6/group); f) The schematic plot of cardiomyocytes cultured with plasma EVs (20 µg/mL) from WT and CC10‐KRAS^G12D^ mice; g) Representative images of immunofluorescence co‐staining showing the uptake of WT‐EVs/CC10‐KRAS^G12D^‐EVs by cardiomyocytes after co‐culturing with Dil‐labelled EVs for 24 h (N = 6/group, Bar: 20 µm). h) Representative images and quantitative results of ROS levels detected by ROS fluorescence staining (N = 4/group, Bar: 40 µm); (i) Mitochondrial membrane potentials detected by JC‐1 staining (Bar: 40 µm) (N = 4/group); j) ATP content was measured by ATP assay (N = 4/group); k) Cardiomyocyte viability was detected by CCK‐8 assay (N = 5/group). Data are expressed as mean ± SEM. ^*^ *p* < 0.05; ^**^ *p* < 0.01; ^***^ *p* < 0.001.

To elucidate the direct detrimental action of lung tumor on cardiac damage, we co‐cultured TC‐1 cells or LLC cells with mouse neonatal cardiomyocytes under normoxia or hypoxia/reoxygenation (H/R) conditions in vitro (**Figure** **4**a). The results showed that cardiomyocytes co‐cultured with LLC cells exhibited significantly higher levels of reactive ROS generation, cTnI release, and LDH release compared to those co‐cultured with TC‐1 cells, under both normoxia and H/R conditions (Figure 4b–d). Additionally, co‐culture with LLC cells significantly reduced cardiomyocyte viability compared to TC‐1 cells, regardless of H/R treatment (Figure 4e). Next, we investigated the effects of Lung cancer‐derived EVs on cardiomyocytes injury. To this end, plasma EVs were isolated from WT and CC10‐KRAS^G12D^ mice and co‐cultured with mouse neonatal cardiomyocytes under H/R condition (Figure 4f). The immunofluorescence staining indicated that DiI‐labeled EVs were internalized by cardiomyocytes within 12 h (Figure 4g). We observed a significant increase in ROS generation in cardiomyocytes incubated with CC10‐KRAS^G12D^‐EVs compared to WT‐EVs under H/R conditions (Figure 4h). To determine whether EVs directly induced metabolic alterations in cardiomyocytes under H/R conditions, mouse neonatal cardiomyocytes were treated with EVs for 24 h. CC10‐KRAS^G12D^‐EVs induced cardiomyocyte metabolism damage under H/R conditions, as demonstrated by significant decreases in mitochondrial Δψm, adenosine 5′ trisphosphate (ATP) synthesis, and cell viability (Figure 4i–k). Furthermore, N‐EVs or C‐EVs were isolated and co‐cultured with mouse neonatal cardiomyocytes under both normoxia and H/R conditions, and these results showed that EVs derived from tumor cells could efficiently fuse with cardiomyocytes, as determined by immunofluorescence staining (Figure S9a,b, Supporting Information). Consistently, similar results were verified by ROS staining, LDH staining, and cell activity assay (Figure S9c–e, Supporting Information). These data show that lung tumor‐secreted plasma EVs promote cardiomyocyte injury.

### 2.5 Lung Cancer Cells‐Derived EVs Derived miR‐485‐3p Promote Cardiac I/R Injury

**Figure 5:**
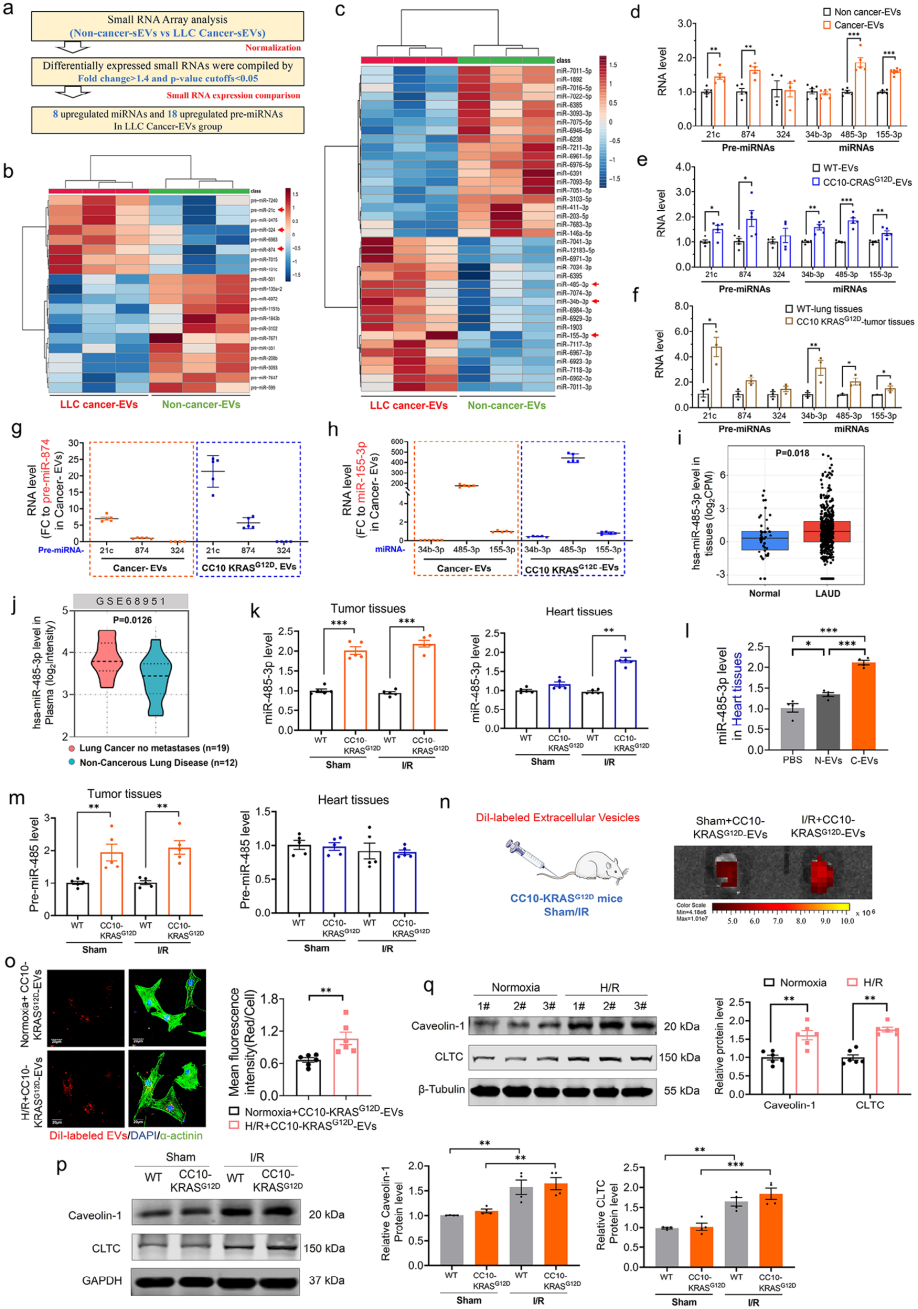
Lung cancer cells‐derived extracellular vesicles transfer miR‐485‐3p from tumor cells to cardiomyocytes a) Schematic presentation of small RNA array analysis of plasma EVs from non‐cancer and LLC lung cancer mice; b‐c) Hierarchical clustering heatmap of differentially expressed of pre‐miRNAs and miRNAs in plasma EVs from non‐cancer and LLC lung cancer mice; d) qRT‐PCR analysis on the expression of pre‐miRNAs and miRNAs with the plasma EVs of non‐cancer and LLC lung cancer mice (N = 4–6/group); e) qRT‐PCR analysis on the expression of pre‐miRNAs and miRNAs with the plasma EVs of WT and CC10‐KRAS^G12D^ mice (N = 4‐5/group); f) qRT‐PCR analysis on the expression of pre‐miRNAs and miRNAs with the lung tissues of WT and CC10‐KRAS^G12D^ mice (N = 3/group); g) The comparisons of pre‐miR‐21c, pre‐miR‐874 and pre‐miR‐324 expression in plasma EVs of cancer mice (N = 4‐5/group); h) The comparisons of miR‐34b‐3p, miR‐485‐3p and miR‐155‐3p expression in plasma EVs of cancer mice (N = 5‐6/group); i) miR‐485‐3p expression in lung adenocarcinoma samples and normal samples from TCGA database (lung adenocarcinoma patient samples, n = 513; normal samples, n = 46); j) TCGA database showed the miR‐485‐3p expression in plasma from patients of lung cancer and non‐cancerous lung disease; k) The expression of miR‐485‐3p with tumor and heart tissues from sham/IR in WT and CC10‐KRAS^G12D^ mice (N = 5/group); l) The expression of miR‐485‐3p in cardiac tissue of intramyocardial injection EVs mice, analyzed by qRT‐PCR (N = 4/group); m) The expression of pre‐miR‐485 with tumor and heart tissues from sham/IR in WT and CC10‐KRAS^G12D^ mice, and the values from IR were normalized to the sham group (N = 5/group); n) Bioluminescence imaging showed that the uptake of CC10‐KRAS^G12D^‐EVs by the heart tissues after I/R. o) Representative images of immunofluorescence co‐staining showing the uptake of CC10‐KRAS^G12D^‐EVs by normoxia or hypoxia/reoxygenation cardiomyocytes after co‐culturing with Dil‐labelled EVs for 24 h (N = 6/group, Bar: 20 µm); p) The protein expression levels of CLTC and Caveolin‐1 were determined by western blot in heart tissues (N = 4/group); q) Western blot detected the protein expression levels of CLTC and Caveolin‐1 in normoxia or hypoxia/reoxygenation cardiomyocytes (N = 6/group). Data are expressed as mean ± SEM. ^*^ *p* < 0.05; ^**^ *p* < 0.01; ^***^ *p* < 0.001.

EVs can fuse with the cytoplasm of target cells to perform regulatory functions.^[^ ^34^ ^]^ Small RNAs are major regulatory components of EVs, we therefore analyzed small RNA contents in plasma EVs derived from cancer versus non‐cancer mice (**Figure** **5**a; Figure S10, Supporting Information). To explore the mechanism underlying cardiac injury‐promoting effect of lung cancer derived EVs, we employed Small RNA expression array to profiling miRNA expression in EVs. The results revealed that 20 pre‐miRNAs (Figure 5b) and 38 miRNAs (Figure 5c) were differentially expressed (FC > 1.4, p‐value < 0.05) in the lung cancer‐derived EVs compared with EVs from the non‐cancer group. Of those, 6 conserved small RNAs across species including pre‐miR‐21c, pre‐miR‐874, pre‐miR‐324, miR‐34b‐3p, miR‐485‐3p, and miR‐155‐3p were chosen as candidates for quantitative validation using qRT‐PCR. The results demonstrated that the expression of pre‐miR‐21c, pre‐miR‐874, miR‐485‐3p, and miR‐155‐3p were significantly higher in both cancer‐EVs and CC10‐KRAS^G12D^‐EVs than in non‐cancer‐EVs and WT‐EVs (Figure 5d,e). Further investigation revealed that levels of pre‐miR‐21c, miR‐34b‐3p, miR‐485‐3p, and miR‐155‐3p were significantly elevated in CC10‐KRAS^G12D^ tumor tissues than in WT lung tissue controls (Figure 5f). Besides, the baseline levels of pre‐miR‐21c and miR‐485‐3p were the most abundant ones in both cancer‐EVs and CC10‐KRAS^G12D^‐EVs compared with others (Figure 5g,h). In addition, the observation that hsa‐miR‐485‐3p is elevated specifically in lung adenocarcinoma (LUAD) and thyroid carcinoma, but not in the other 31 tumors analyzed in the Cancer Genome Atlas (TCGA) miRNome (https://bioinfo.jialab-ucr.org/CancerMIRNome/) database, suggests a potential tissue‐ or cancer type‐specific role for this miRNA (Figure S11, Supporting Information; Figure 5i). Consistently, plasma hsa‐miR‐485‐3p level is remarkably increased in lung cancer patients compared with the patients with non‐cancerous lung disease (Figure 5j).

Moreover, we compared miR‐485‐3p levels in heart and tumor tissues of WT and CC10‐KRAS^G12D^ mice subjected to sham or I/R surgery. The results showed that compared with normal lung tissue in WT group, miR‐485‐3p expression was upregulated in lung tumor tissues of CC10‐KRAS^G12D^ mice (Figure 5k). MiR‐485‐3p expression level was not changed in I/R hearts as compared with sham‐operated controls in the absence of lung tumor. Interestingly, the expression of miR‐485 in the heart tissue was significantly increased in I/R‐CC10‐KRAS^G12D^ mice compared with WT controls, while this effect was absent in sham‐treated mice heart (Figure 5k). Consistently, tail vein injection of C‐EVs significantly increased cardiac tissue levels of mature miR‐485‐3p (Figure 5l). The levels of miR‐485‐3p were ≈1.67‐fold higher in heart tissues than in tumor tissues of WT sham‐operated mice, and 2.21‐fold higher in heart tissues of CC10‐KRAS^G12D^ mice as compared with WT‐controls (Figure S12a, Supporting Information). To clarify whether the increase of miR‐485‐3p level could be ascribed to its release from tumor cells via EVs or due to its production from cardiomyocytes per se after cancer, we quantified precursor miR‐485 (pre‐miR‐485) expression levels. In CC10‐KRAS^G12D^ mice, pre‐miR‐485 expression was elevated in tumor tissues under both sham and I/R‐operated conditions compared to WT controls, but remained unchanged in heart tissues (Figure 5m). These results suggest that EVs‐packaged miR‐485‐3p secreted by lung tumors mediates its accumulation in myocardial tissue. To clarify whether the elevated miR‐485‐3p levels resulted from increased EVs secretion by tumor tissues under I/R conditions or enhanced cardiac EVs uptake post‐I/R, we measured the expression of Ras‐associated protein 27a (Rab27a), a protein critical for regulating EVs release from organ tissue.^[^ ^35^ ^]^ The results revealed significantly higher Rab27a expression in lung tumor tissues of CC10‐KRAS^G12D^ mice compared to WT lung tissues under both sham and I/R conditions. However, Rab27a expression showed no significant difference between I/R and sham conditions (Figure S12b, Supporting Information). Moreover, bioluminescence imaging demonstrated significantly enhanced cardiac uptake of DiI‐labelled CC10‐KRAS^G12D^‐EVs (4 × 10^9^) in I/R‐treated hearts compared to sham controls following tail vein injection (Figure 5n). Consistent with in vivo result, H/R‐treated cardiomyocytes internalized much more EVs than normoxic group (Figure 5o). EVs uptake primarily occurs via endocytosis, mediated through both Clathrin‐dependent pathway and ‐independent pathway mainly mediated by Caveolin‐1.^[^ ^36^ ^]^ We therefore examined the expressions of Clathrin light chain C (CLTC) and Caveolin‐1 in heart tissues of WT and CC10‐KRAS^G12D^ mice under sham or I/R conditions. Both CLTC and caveolin‐1 expression were significantly upregulated in I/R‐treated hearts compared to sham mice (Figure 5p). Similarly, we found that the higher expression levels of CLTC and Caveolin‐1 in H/R cardiomyocytes compared to normoxic cardiomyocytes (Figure 5q). Collectively, these findings demonstrate that tumor‐derived EVs promote miR‐485‐3p accumulation in cardiac tissue through enhanced endocytic uptake, ultimately exacerbating I/R‐induced myocardial injury.

**Figure 6:**
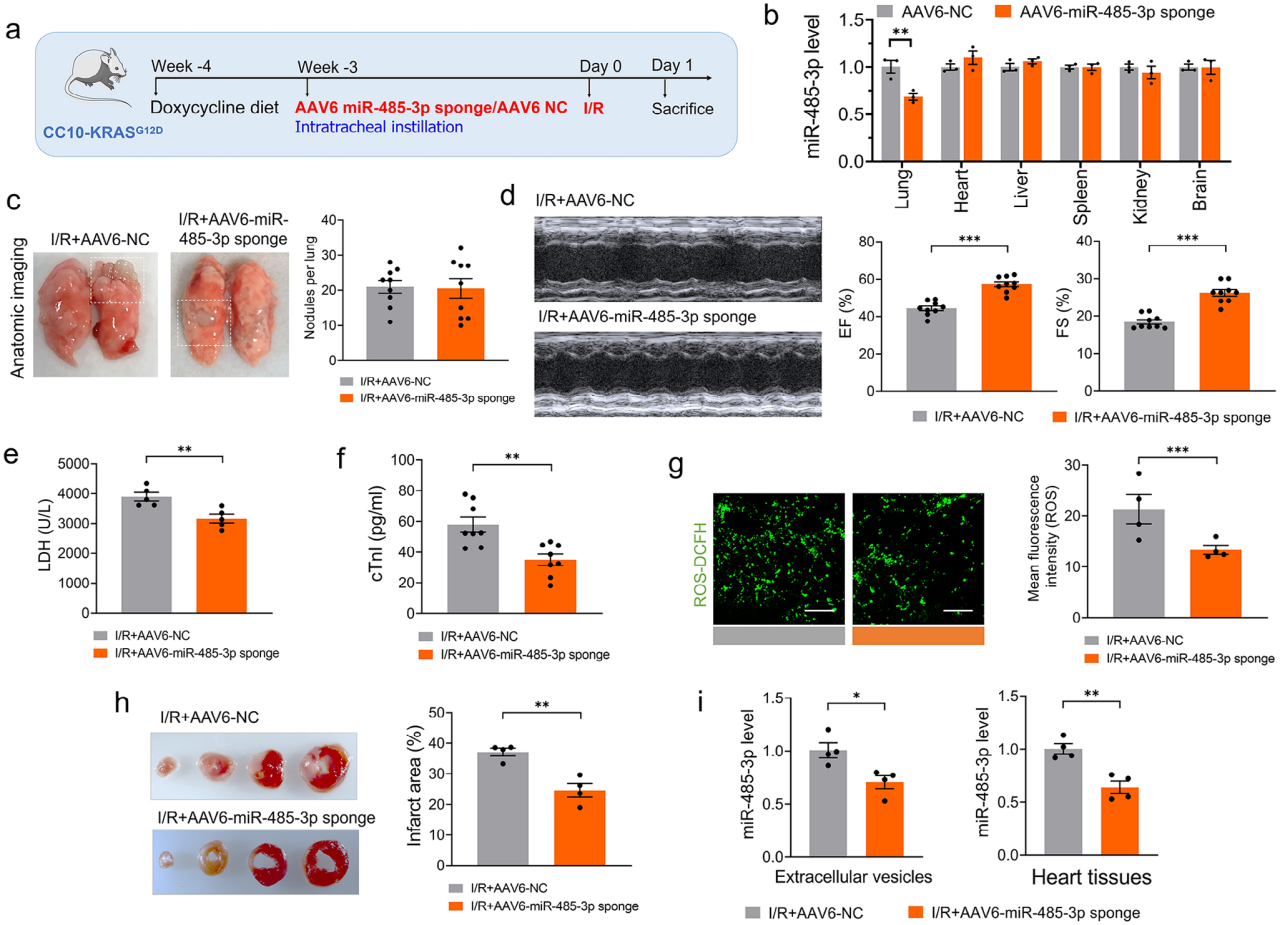
Lung cancer cells‐derived EVs derived miR‐485‐3p promote cardiac I/R injury a) Schematic presentation on the time‐line of in vivo animal model construction experiment in CC10‐KRAS^G12D^ mice treated with AAV6‐miR‐485‐3p sponge/NC, CC10‐KRAS^G12D^ mice were treated with ≈0.1 mg kg^−1^ doxycycline daily. After 7 days, adeno‐associated virus (AAV) 6 with spc promoter mediated miR‐485‐3p sponges/NC to the lung were injected into mice via intratracheal instillation. After 3 weeks, the mice were subjected to I/R operation for 24 h; b) qRT‐PCR analysis on the expression of miR‐485‐3p in lung, heart, liver, spleen, kidney and brain tissues (N = 3/group); c) Representative anatomic images and statistics analysis of lung tumors (N = 9/group); d) Representative images of echocardiographs and statistical data on EF%, FS% (N = 9/group); e,f) Plasma LDH (N = 5/group) and cTnI (N = 8/group) levels were detected in mice; g) Representative images and quantitative results of ROS levels detected by ROS fluorescence staining (Bar: 40 µm) (N = 4/group); h) Infarct area of I/R mice by TTC staining (N = 4/group); i) qRT‐PCR analysis on the expression of miR‐485‐3p in plasma extracellular vesicles and heart tissues from CC10‐KRAS^G12D^ mice treated with AAV6‐miR‐485‐3p sponge/NC (N = 4/group). Data are expressed as mean ± SEM. *P < 0.05; ^**^ *p* < 0.01; ^***^ *p* < 0.001.

To further investigate the role of miR‐485‐3p‐containing extracellular vesicles in cardiac function in vivo, adeno‐associated virus (AAV) 6 with spc promoter mediated miR‐485‐3p sponges/NC were injected into the lung of CC10‐KRAS^G12D^ mice as described earlier, mice were subjected to I/R surgery after 3 weeks (**Figure** **6**a). AAV6‐mediated delivery of miR‐485‐3p sponges significantly reduced miR‐485‐3p levels in lung tissues (*p*<0.01) but not in other organs (liver, spleen, kidney, or brain) compared to NC‐treated mice, confirming the specificity of the knockdown (Figure 6b). No significant difference in total tumor burden was observed between AAV6‐miR‐485‐3p sponge mice and AAV6‐NC mice groups (Figure 6c). Inhibition of miR‐485‐3p exhibited a pronounced alleviation in I/R‐induced decreases in EF% and FS% (Figure 6d). Furthermore, I/R‐induced cardiac damage was largely attenuated by knockdown of miR‐485‐3p, as evidenced by reductions in plasma LDH and cTnl levels, and ROS production (Figure 6e–g). Consistent with this finding, myocardial infarct size was significantly smaller in AAV6‐miR‐485‐3p sponge‐treated mice than in AAV6‐NC mice (Figure 6h). Notably, with I/R, miR‐485‐3p expression was significantly decreased in both EVs and heart tissues in AAV6‐miR‐485‐3p sponge mice compared with AAV6‐NC mice (Figure 6i). Additionally, no significant differences were observed in the expression levels of miR‐34b‐3p and miR‐155‐3p in either EVs or heart tissues (Figure S13, Supporting Information).

### 2.6 MiR‐485‐3p Triggers Cardiomyocyte Death by Targeting PGC‐1α

**Figure 7:**
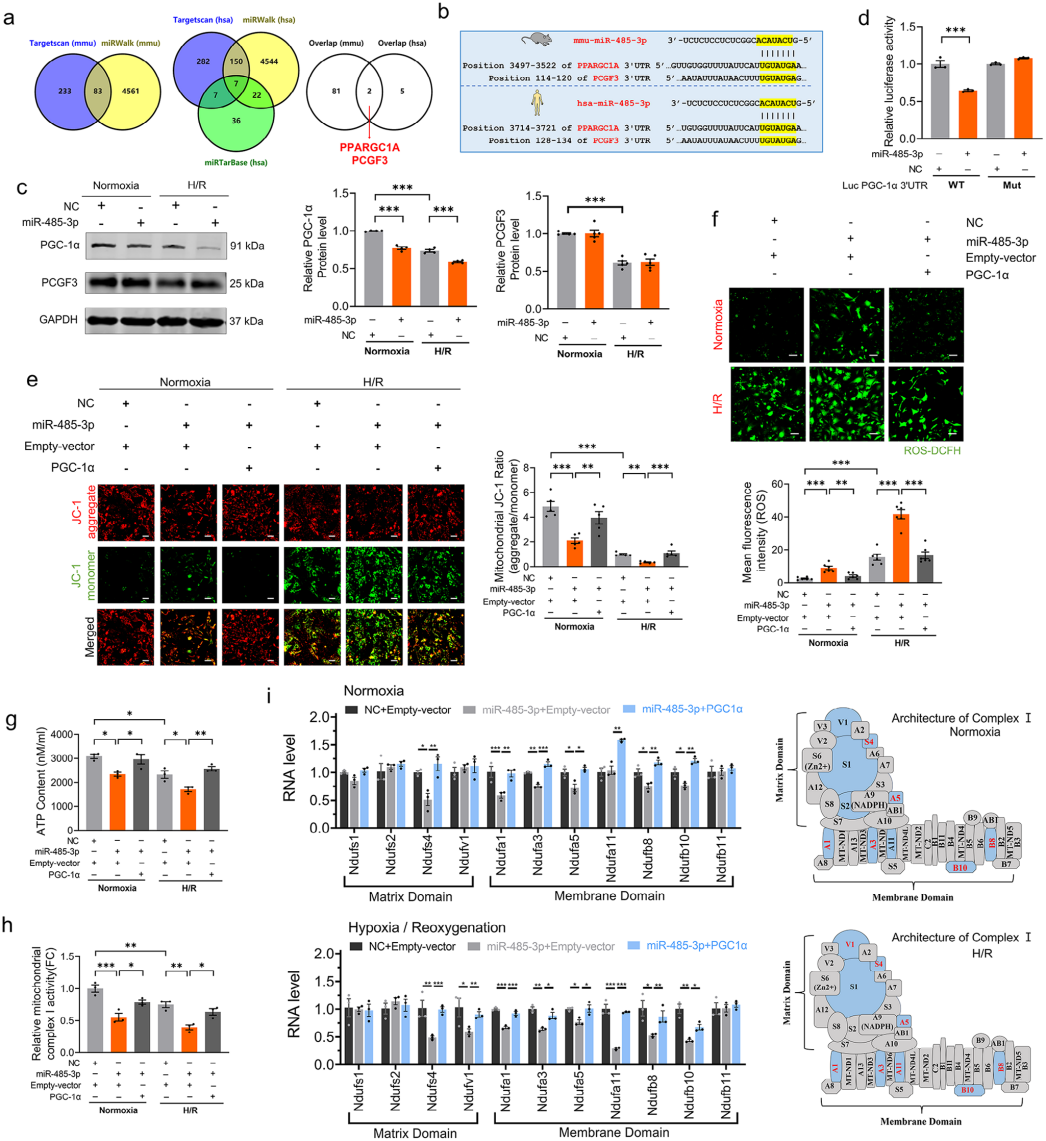
MiR‐485‐3p triggers cardiomyocyte death by targeting PGC‐1α to impair mitochondrial complex I activity in H/R model in vitro a) The candidate target genes of miR‐485‐3p were searched with Targetscan, miRWalk and miRTarBase database; b) The sequence of miR‐485‐3p and the potential binding site of PCGF3 mRNA; c) The protein expression levels of PGC‐1α and PCGF3 were determined by western blot (N = 4‐5/group); d) Luciferase activity assay was performed to confirm the PGC‐1α mRNA was directly bound to miR‐485‐3p in HEK‐293T cells (N = 3/group); e) Mitochondrial membrane potentials detected by JC‐1 staining (Bar: 40 µm); f) Representative images and quantitative results of ROS using ROS fluorescence staining (Bar: 40 µm); g) ATP content was measured by ATP assay (N = 3/group); h) The activity of mitochondrial complex I was detected by ELISA assay (N = 3/group); i) The expression of ETC complex I genes Ndufs1, Ndufs2, Ndufs4, Ndufv1, Ndufa1, Ndufa3, Ndufa5, Ndufa11, Ndufb8, Ndufb10 and Ndufb11 in myocardial cells that were stimulated with NC/miR‐485‐3p mimics or empty‐vector/PGC‐1α under normoxia or hypoxia/reoxygenation conditions (N = 3/group). Data are expressed as mean ± SEM. ^*^ *p* < 0.05; ^**^ *p* < 0.01; ^***^ *p* < 0.001.

To uncover the underlying mechanism by which miR‐485‐3p promotes cardiomyocyte damage, we searched for candidate target genes of miR‐485‐3p by computational prediction with TargetScan (https://www.targetscan.org/vert_80/), miRWalk (http://mirwalk.umm.uni-heidelberg.de/), and miRTarBase (https://mirtarbase.cuhk.edu.cn/%7E;miRTarBase/miRTarBase_2022/php/index.php). We identified that two genes, peroxisome proliferator‐activated receptor gamma coactivator 1‐alpha (PPARGC1A) and polycomb group ring finger 3 (PCGF3), might be the potential targets of miR‐485‐3p in overlap between mouse and human sapiens (**Figure** **7**a). PPARGC1A and PCGF3 contained one conserved 8‐mer site (Figure 7b). The CancerMIRNome analysis shows the miR‐485‐3p and PPARGC1A expression low‐correlation in lung adenocarcinoma tissues (Figure S14, Supporting Information). To establish a possible effect of miR‐485‐3p on the expression of the two proteins expressed in cardiomyocytes, we analyzed their levels after miR‐485‐3p overexpression under normoxia and H/R conditions. We observed that overexpression of miR‐485‐3p did not affect PCGF3 protein expression while markedly decreased the protein level of PGC‐1α (encoded by the PPARGC1A gene) as compared to control NC transfected cells (Figure 7c). We next performed luciferase reporter assays to verify that miR‐485‐3p repressed luciferase activity after co‐transfecting with the PGC‐1α 3′‐UTR containing luciferase reporter constructs but not its mutated constructs (Figure 7d). It is well known that PGC‐1α regulates the mitochondrial and energy metabolism homeostasis via acting as the co‐activating several transcription factors (TFs) to stimulate the RNA polymerase II (RNAPII) dependent transcription of target genes.^[^ ^37^ ^]^ To determine whether miR‐485‐3p directly caused cardiomyocyte injury under normoxia and H/R conditions, mouse neonatal cardiomyocytes were stimulated by miR‐485‐3p mimics for 24 h. Overexpression of miR‐485‐3p caused cardiomyocyte damage with or without H/R treatment, as reflected by striking reductions in mitochondrial Δψm, ATP synthesis, and increase in ROS generation, whereas these effects were markedly reversed by co‐transfection of PGC‐1α overexpression (Figure 7e‐g). Mitochondrial ETC complex I is the first enzyme complex of the ETC, which couples with transmembrane proton (H^+^) pumping, which generates Δp necessary for ATP synthesis.^[^ ^38^ ^]^ In line with the findings presented above, the activity of ETC complex I was decreased by miR‐485‐3p overexpression, which was reversed by co‐transfection of PGC‐1α overexpression in cardiomyocytes under normoxia and H/R conditions (Figure 7h). To further corroborate this observation, the gene expressions of mitochondrial ETC complex I were assessed. As shown in Figure 7i, we found that the decreased expression of ETC complex I subunits Ndufa1, Ndufa3, Ndufa5, Ndufa11, Ndufb8, Ndufb10, Ndufs4, Ndufv1 induced by miR‐485‐3p overexpression were attenuated by PGC‐1α overexpression in cardiomyocytes under normoxia and H/R conditions. The loss of any subunits to the integrated structure of ETC complex I, which leads to mitochondrial dysfunction.^[^ ^39^ ^]^

**Figure 8:**
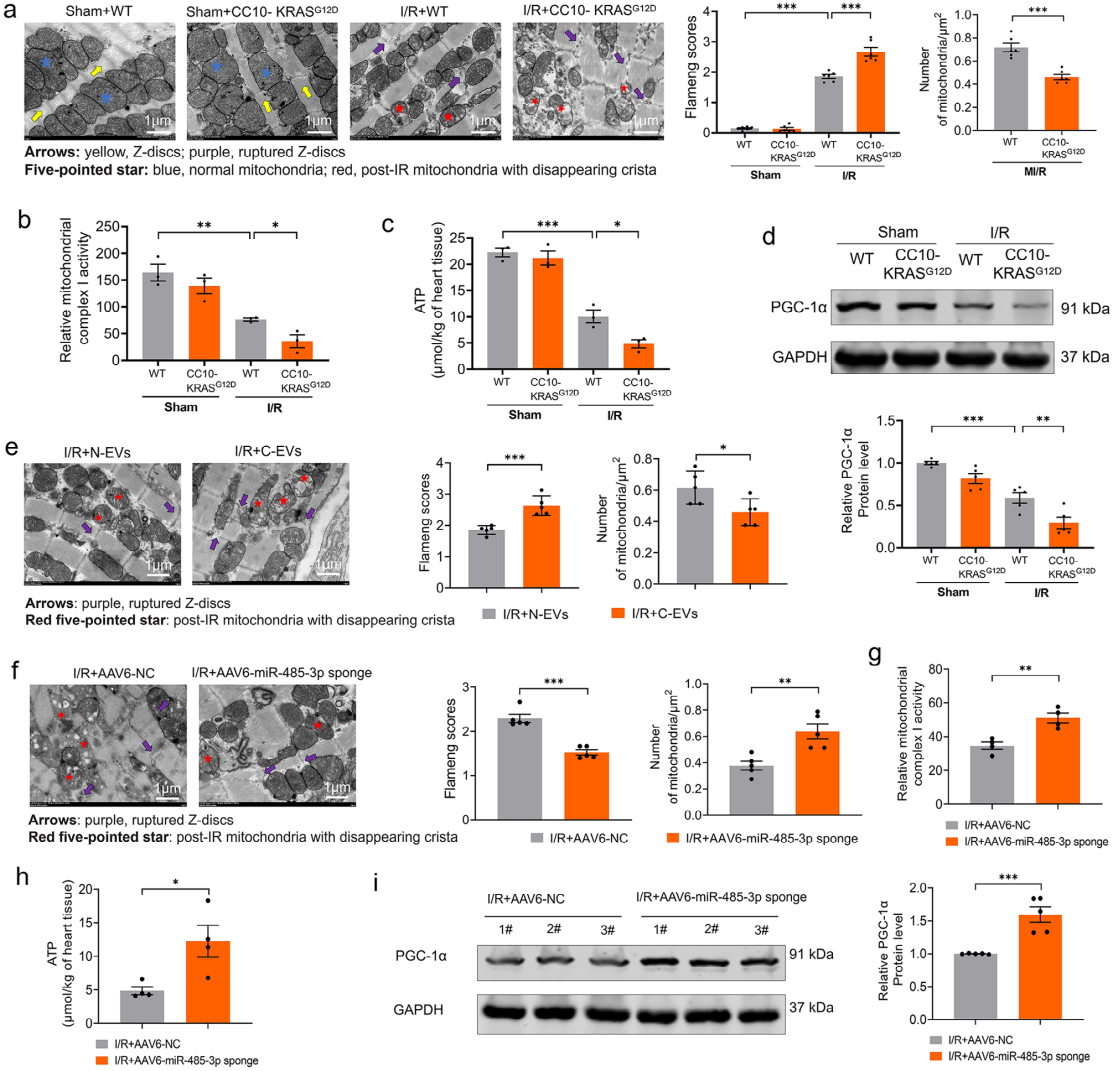
MiR‐485‐3p enhances mitochondrial dysfunction after I/R injury by targeting PGC‐1α in vivo a) Representative transmission electron microscopy images in heart of WT and CC10‐KRAS^G12D^ mice (Bar: 1 µm), mitochondria were quantitatively analyzed using the ImageJ software, number of mitochondria/µm^2^ refers to the average number of mitochondria (N = 3/group, 6 statistical images from three mice); b) The activity of mitochondrial complex I was detected by ELISA assay (N = 3/group); c) ATP content was measured by ATP assay (N = 3/group); d) The protein expression levels of PGC‐1α were determined by western blot (N = 5/group); e) Representative transmission electron microscopy images in heart of N‐EVs and C‐EVs mice with I/R (Bar: 1 µm), mitochondria were quantitatively analyzed using the ImageJ software, number of mitochondria/µm^2^ refers to the average number of mitochondria (N = 4/group); f) Representative transmission electron microscopy images in heart of AAV6‐miR‐485‐3p sponge and NC CC10‐KRAS^G12D^ mice with I/R (Bar: 1 µm), mitochondria were quantitatively analyzed using the ImageJ software, number of mitochondria/µm^2^ refers to the average number of mitochondria (N = 3/group, 5 statistical images from three mice); g) The activity of mitochondrial complex I was detected by ELISA assay (N = 4/group); h) ATP content was measured by ATP assay (N = 4/group); (i) Quantification and representative images for PGC‐1α by western blot (N = 5/group). Data are expressed as mean ± SEM. ^*^ *p* < 0.05; ^**^ *p* < 0.01; ^***^ *p* < 0.001.

Transmission electron microscope results revealed no significant differences in mitochondrial morphology in heart tissue between WT and CC10‐KRAS^G12D^ mice. However, following I/R injury, both groups exhibited severe mitochondrial structural disruption and impaired biosynthesis compared to sham‐operated controls, with these pathological changes being more pronounced in CC10‐KRAS^G12D^ mice (**Figure** **8**a). In line with in vivo result, the activity of ETC complex I, ATP synthesis and PGC‐1α expression were reduced in cardiac mitochondria in CC10‐KRAS^G12D^ mice as compared with WT mice, and these effects were further decreased with I/R model (Figure 8b–d). C‐EVs treated mice exhibited a significantly greater exacerbation of mitochondrial damage and impaired biosynthesis in mouse heart compared with N‐EVs treated mice following I/R injury (Figure 8e). As shown in Figure 8f, AAV6‐miR‐485‐3p sponge‐infected mice showed a significant improvement in mitochondrial damage and biosynthesis in mouse heart compared with AAV6‐NC treated mice following I/R injury. Furthermore, AAV6‐miR‐485‐3p sponge reversed I/R induced reductions of the activity of ETC complex I, ATP synthesis and PGC‐1α expression compared with AAV6‐NC in CC10‐KRAS^G12D^ mice (Figure 8g–i). These findings demonstrate that miR‐485‐3p exacerbates I/R injury by directly targeting PGC‐1α in cardiomyocytes.

### 2.7 Study Limitations

First, we synthesized the evidence regarding mortality outcomes in patients with versus without cancer undergoing PCI through a systematic review and meta‐analysis. However, in light of the increasing number of patients with both IHD and cancer, current understanding and the management of IHD in cancer patients to evolve, and more data, information, and considerations presented were included in the analysis in the future, as more rigorous data and new hypotheses come up. Second, despite substantial evidence supporting that chemotherapy and radiotherapy can cause cardiotoxicity, cardiac wasting has been reported clinically in cancer patients before initiating any anticancer therapy.^[^ ^5^, ^6^ ^]^ A previous study reported that breast cancer induced‐cardiac injury, LVEF, and LVFS remained unchanged in mice, cardiac morphology, LV mass, GLS, GLSR, GRS, GRSR, LVPWd, and LVPWs were decreased at 16 weeks. In our cancer model mice, we didn't pay much attention to whether prolonging bearing cancer causes cardiac dysfunction. Third, the methods of infusing EVs in experimental animals or adding them to cell cultures have been questioned. The administration of exogenous EVs has the limitation of not fully replicating the natural release of EVs. However, given that lung cancers secrete multiple additional factors (e.g., HSPA1A, MFGE8, ANXA4),^[^ ^40^ ^]^ our approach allows us to study and dissect the role of tumor‐derived EVs independent of other secreted factors. Finally, although our results suggest a significant contribution of tumor cell‐EVs to I/R cardiac injury, we acknowledge the need to investigate the role of other EVs contents of tumor cells or EVs of other cells from tumor in I/R cardiac injury.

## 3 Discussion

**Figure 9:**
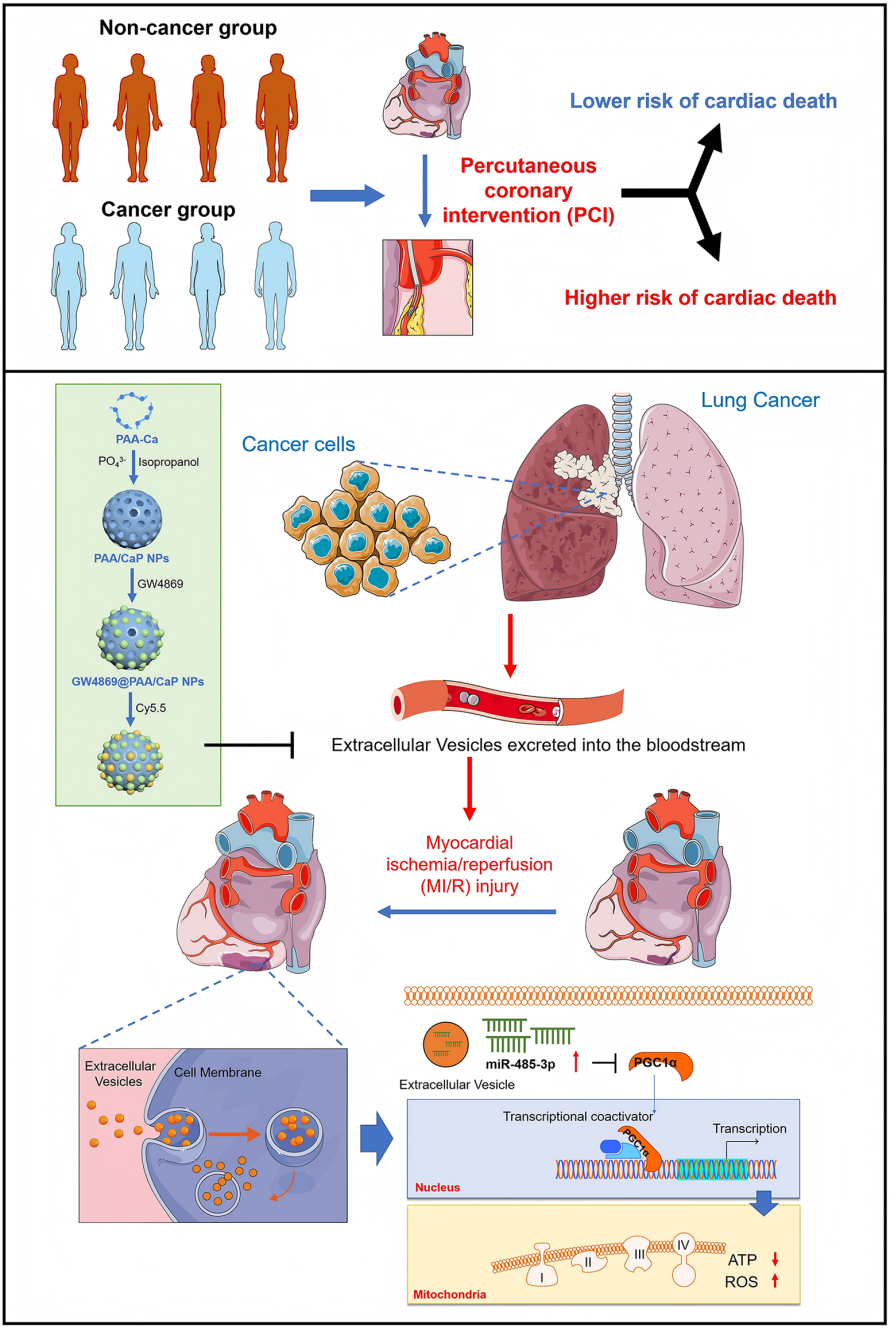
Schematic illustration on the proposed signaling pathway linking between the cancer and I/R cardiac injury.

It is known that circulating factors may explain the interactions of the relationship between CVDs and cancer, two distinct disease entities. But the mechanisms are indeed complex, and we are still facing many challenges in this new cardio‐oncology field. Here, for the first time, we demonstrate a mechanistic link between cancer and I/R cardiac injury (**Figure** **9**).

Cardiologists are encountering a growing number of patients with both ischemic heart disease (IHD) and cancer. In the present meta‐analysis, we have shown that patients with cancer have greater all‐cause mortality, including cardiovascular mortality and non‐cardiovascular mortality. Noteworthy, cancer patients had a higher risk of cardiovascular mortality within 1 year compared with those patients over 1 year.^[^ ^25^ ^]^ Although this finding was not reported in the majority of the studies analyzed and thus not included in our analysis. In addition, another study found that cancer patients were close to a six‐fold increased risk of non‐cardiovascular mortality at one‐year after PCI compared to non‐cancer patients.^[^ ^41^ ^]^ It is likely that mortality due to malignancy itself is the main variable leading to increased one‐year non‐cardiovascular mortality and subsequently increased all‐cause mortality at one‐year in the cancer group. A growing body of evidence indicates that the interaction between IHD and cancer is complex, influenced not only by biological processes but also by some antineoplastic drugs. Some of these medications have been associated with a higher risk of CV mortality undergoing PCI.^[^ ^7^ ^]^ Emerging data indicate that acute myocardial infarction (AMI) and coronary revascularization are 1.5–3 times more likely in oncological patients receiving immune checkpoint inhibitors than in the placebo arms of RCTs or in controls of observational investigations.^[^ ^42^, ^43^ ^]^

Mitochondrial disorders causing ROS accumulation, energy stress, and cell death are associated with a high incidence of cardiac involvement.^[^ ^44^, ^45^ ^]^ However, the mechanism by which cancer induces cardiomyocyte mitochondrial dysfunction remains complex and poorly understood. An intriguing new finding in the present study is that CC10‐KRAS^G12D^ cancer mice aggravated metabolism change of cardiomyocytes after I/R, mediated by suppression of mitochondrial complex I activity and ATP synthesis. Elevated levels of miR‐485‐3p in plasma extracellular vesicles implicate inhibition of PPARGC1A/PGC‐1α as a key driver of maintaining mitochondrial and energy metabolism homeostasis. In conclusion, the lung cancer‐derived EVs enriched with miR‐485‐3p induce persistent mitochondrial damage effect in cardiomyocytes.

Both EF% and FS% were significantly decreased after I/R as compared with sham‐operated mice, and these effects were further decreased in cancer combined with I/R model (Figure 1f,g). However, cancer alone did not induce cardiac dysfunction compared to non‐cancer controls. A previous study reported that breast cancer induced‐cardiac injury, LVEF and LVFS remained unchanged in mice. Cardiac morphology, LV mass, GLS, GLSR, GRS, GRSR, LVPWd, and LVPWs were decreased in 16‐week‐old MMTV‐PyMT(+) mice compared with 16‐week‐old MMTV‐PyMT(–) mice. And cardiac fibrosis was significantly more obvious in 16‐week‐old MMTV‐PyMT(+) mice. But cardiac dysfunction remained unchanged at 12‐weeks.^[^ ^46^ ^]^ Consistent with these reports, our study did not detect cancer‐induced cardiac dysfunction at the 6‐week endpoint, suggesting that this duration may be insufficient for malignancy to exhibit significant cardiac damage under non‐IR conditions. In addition, a proteomic study of non‐ NSCLC cells‐derived EVs identified HSPA1A, MFGE8, and ANXA4 as mediators of tumor‐recipient cells crosstalk.^[^ ^40^ ^]^ While these molecules could play roles in other contexts, our experimental evidence strongly supports the dominance of miR‐485‐3p in this specific model. Nevertheless, we cannot entirely exclude the possibility of minor contributions from other EV constituents. This is something that we need to explore further in the future.

Several studies have revealed the complex roles of miR‐485‐3p in cancers. Treatment with miR‐485‐3p siRNAs reduced tumor growth, invasion, and metastasis in an orthotopic liver cancer model.^[^ ^47^ ^]^ Another study showed that miR‐485‐3p had potent sensitizing activity with doxorubicin in multiple models of high‐risk neuroblastoma.^[^ ^48^ ^]^ In our study, AAV6‐mediated miR‐485‐3p sponges/NC to the lung were injected into the CC10‐KRAS^G12D^ mice for 3 weeks; however, we observed no significant difference in tumor burden between the two groups. Interestingly, in line with our findings, a separate study showed that miR‐485‐3p suppresses breast cancer cell metastasis by inhibiting PGC‐1α expression.^[^ ^49^ ^]^ It is known that heart specific PGC‐1α knock‐out induces metabolic, functional and structural abnormalities leading to dilating cardiomyopathy and heart failure.^[^ ^50^ ^]^ Notably, we provide the first evidence that miR‐485‐3p levels are elevated in the plasma of CC10‐KRAS^G12D^ cancer model mice, and that tumor‐derived extracellular vesicles containing miR‐485‐3p exacerbate ischemia/reperfusion‐induced cardiac injury. Additionally, another study has also revealed the role of miR‐485‐3p in the exosomal secretion.^[^ ^51^ ^]^ We demonstrated that miR‐485‐3p levels were significantly increased in tumor cell‐derived plasma extracellular vesicles. Moreover, miR‐485‐3p was significantly increased in tumor, heart and plasma extracellular vesicles in cancer mouse model, whereas pre‐miR‐485 was increased only in the tumor tissue, which indicated that miR‐485‐3p was produced by the tumor cells and carried by extracellular vesicles from the tumor to heart. Notably, cardiac injury mediated by cancer‐derived extracellular vesicles occurred only in I/R mice, which correlated with enhanced cardiac EVs uptake post‐I/R. Extracellular vesicle uptake has been reported to occur through multiple routes that involve endocytosis by either clathrin‐dependent or clathrin‐independent pathways.^[^ ^36^ ^]^ Clathrin‐mediated endocytosis is a key process in extracellular vesicles uptake, transporting a wide range of cargo molecules from the cell surface to the interior. Clathrin‐independent endocytosis primarily involves caveolin‐1, RhoA, and ARF6,^[^ ^52^ ^]^ caveolins can mediate the formation of specific microdomains within the plasma membrane, also known as lipid rafts, which serve as entry points for extracellular vesicles. We found that the higher expression levels of Clathrin and Caveolin‐1 in the heart tissues of I/R mice compared to sham mice.

Using polyacrylic acid (PAA) as a template for calcium phosphate nanoparticles (CaP NPs) synthesis offers several advantages, including a simple and scalable synthetic route, a high GW4869 payload, and dual pH‐responsive drug release. Therefore, PAA/CaP@NPs represent a promising drug delivery system for enhancing the therapeutic efficacy of anticancer drugs. However, over the past decade, continuous development and investigation of new drug delivery systems with diverse morphologies and structures have made great progress. For example, nano‐sized MnO_2_ exhibits reduced particle size, increased surface area, and enhanced catalytic properties, making it a promising theranostic material for tumors.^[^ ^53^ ^]^ Similarly, Zhang et al. reported that a drug delivery vehicle can be preferentially internalized by circulating monocytes and induce their transformation into an anti‐inflammatory phenotype upon reaching the inflammation site, thereby improving therapeutic outcomes.^[^ ^54^ ^]^

In our study, to induce mouse tumor model, the CC10‐KRAS^G12D^ mice were fed normal food pellets supplemented with doxycycline, that is, these WT mice and bitransgenic mice were treated with ≈0.1 mg kg^−1^ doxycycline daily for 4 weeks. In fact, studies have suggested that early short‐term doxycycline treatment ameliorates post‐infarction remodeling and exerts protective effects on myocardial ischemia/reperfusion injury in experimental^[^ ^55^, ^56^ ^]^ and clinical settings.^[^ ^57^ ^]^ Doxycycline is less toxic, and doxycycline (30 mg kg^−1^ twice daily orally for 2 weeks) was able to attenuate the LV cardiomyocyte hypertrophy.^[^ ^58^ ^]^ In contrast, the dose of 0.1 mg kg^−1^ was much lower than the dose that improved cardiac remodeling. Consistent with the low toxicity of doxycycline, after 4 weeks of doxycycline diet induction, histopathological analysis revealed no inflammatory infiltrates or structural abnormalities in the heart, liver, spleen, kidneys, or brain of CC10‐KRAS^G12D^ mice.

In summary, tumor‐secreted extracellular vesicles promote I/R cardiac injury. The present study demonstrates that mature miR‐485‐3p levels are significantly increased in lung cancer cells. MiR‐485‐3p is packaged into extracellular vesicles and transferred from tumor cells to cardiomyocytes, where it represses the expression of PGC‐1α, a critical regulator of the mitochondria and energy metabolism homeostasis. Lung‐specific block of miR‐485‐3p biogenesis or promotion of target gene PGC‐1α activation may be a novel effective therapeutic avenue to block EVs‐mediated pathological communications between I/R heart and tumor tissues and heart tissues.

## 4 Experimental Section

The data that support the findings are available from the corresponding author upon reasonable request. All animal experimental protocols were approved by Ethics Committee of Harbin Medical University (IRB3035724) and conformed to the Guide for the Care and Use of Laboratory Animals published by the US National Institutes of Health.^[^ ^59^ ^]^

### Full Methods for the Systematic Review and Meta‐Analysis

The PubMed and EMBASE databases were searched using the terms “percutaneous coronary intervention” and “cancer” from inception through 1^st^ July 2024. A total of 4689 abstracts were screened, and the relevant articles were reviewed if deemed eligible. The reference lists of these publications were also checked in order to identify potential additional studies of interest, which were not obtained from the initial database search.

The articles were selected that satisfied the following inclusion criteria: 1) RCTs, registries, or observational studies of patients with CVDs; 2) comparing mortality outcomes between patients with and without cancer; 3) with reported mortality events; and 4) in English language. In the case of overlapping studies, the one with the most cancer patients was included. The full text of the selected articles was read and the following data were extracted: number of participants (n ≥ 100), key characteristics, and outcomes of interest. The quality of the retrieved studies were evaluated by the standardized Newcastle‐Ottawa Scale, where studies with scores of less than 4 were considered to have a high risk of bias, those with scores of 4 to 6 an intermediate risk of bias, and those with scores of 7 or more a low risk of bias.

The Preferred Reporting Items for Systematic Reviews and Meta‐Analysis (PRISMA) guidelines were applied and the incidence rate ratios (IRRs) were calculated along with 95% confidence intervals (95% CIs) for all‐cause and CV mortality.

All analyses were performed with the R statistical software (R project for statistical computing 4.2.1 version, Vienna, Austria) using the R package “meta” and “metafor.” Statistical significance was set at p < 0.05.

### Animals

C57BL/6J mice (6‐8 weeks old, 18–22 g) were obtained from Changsheng Biotechnology Company (China). Food and water were freely accessible by the mice. Neonatal mice (1‐2 days post‐birth) were provided by the Animal Center at the Second Affiliated Hospital of Harbin Medical University. Mice were fed in a facility with 12 h light / 12 h dark cycle at 23 ± 3 °C and 30–70% humidity. All animal experimental protocols were approved by Ethics Committee of Harbin Medical University and conformed to the Guide for the Care and Use of Laboratory Animals published by the US National Institutes of Health. Randomization and blinding were adopted. Briefly, the mice were allocated to experimental groups randomly. Echocardiography measurement and cardiac ischemic and reperfusion injury model were performed by a single experienced operator in a blinded fashion.

### Generation of Plasmid Constructs and Transgenic Mice

The TetO‐KRAS^G12D^ transgene mice and the CC10‐rtTA tetracycline‐dependent activator mice were obtained from VIEWSOLID Biotechnology Company (Beijing, China). The TetO‐KRAS^G12D^ transgene mice were constructed as described previously.^[^ ^60^ ^]^ The CC10‐rtTA tetracycline‐dependent activator mice were produced as described previously.^[^ ^61^ ^]^ Bitransgenic mice were produced by crossing the CC10‐rtTA activator mice to TetO‐KRAS^G12D^ responder mice. To induce mouse tumor model, the mice were fed normal food pellets supplemented with doxycycline (625 mg kg^−1^, Special Diets Services, Konoscience, China) for four weeks starting at the age of two weeks. In other words, these bitransgenic mice were treated with ≈0.1 mg kg^−1^ doxycycline daily for 4 weeks.

### PCR Genotyping

Tail DNA was isolated using Qiaprep Tail DNeasy isolation kit (QIAGEN) according to the manufacturer's protocol. Detection of the CC10‐rtTA activator transgene and the TetO‐KRAS^G12D^ transgene were done using the following primers:

CC10‐rtTA: F 5′‐AAAATCTTGCCAGCTTTCCCC‐3′ and

CC10‐rtTA: R 5′‐ACTGCCCATTGCCCAAACAC‐3′ (generates a 500 bp product);

TetO‐KRAS^G12D^: F 5′‐AGACACAAAACAGGCTCAGGA‐3′ and

TetO‐KRAS^G12D^: R 5′‐GGAGACAATGGTTGTCAACAGA‐3′ (generates a 400 bp product).

Reactions were amplified with the following PCR protocol: 94 °C denaturation for 2 min, followed by 35 cycles of 98 °C for 10 s, 60 °C for 30 s, 68 °C for 60 s, followed by a 10 min extension at 68 °C and 2 min 16 °C. PCR products were resolved on a 2% agarose gel.

### Mouse Model of Myocardial I/R Injury

The mice were anesthetized with Avertin (0.2 g kg^−1^ i.p., Sigma–Aldrich, St Louis, USA) and connected to a ventilator (Comerio, ITALY) via tracheal intubation. After exposing the heart, the left anterior descending coronary artery was ligated at 2 mm below the left atrium for 45 min with a 7/0 nylon suture and thereafter released for a 24 h/7 days period for reperfusion. Sham‐operated mice were treated by the same procedures but without LAD ligation.

### Infection of Adeno‐Associated Virus Carrying miR‐485‐3p Sponge

The (AAV) 6 vectors with the pulmonary epithelial cells‐specific spc promoter carrying miR‐485‐3p sponge and negative control (NC) were constructed by HANbio biotechnology (China). The AAV6 virus (2.0 × 10^11^ genomes per mouse) was delivered into CC10‐KRAS^G12D^ mice by intratracheal instillation for 3 weeks before I/R surgery.

### Determination of Cardiac Troponin I (cTnI) and Lactate Dehydrogenase (LDH) Levels

The cTnI and LDH concentrations of mouse plasma were measured using a cTnI Assay Kit (E‐EL‐M1203c, Elabscience, China) and a LDH Assay Kit (A095‐2‐1, Jiancheng Bioengineering Institute, China). Briefly, mouse hearts were subjected to 45 min of ischemia followed by reperfusion at different durations (24 h or 7 days), and plasma samples were collected accordingly. In addition, to investigate the direct detrimental effects of lung tumors on cardiac injury, TC‐1 cells or LLC cells were co‐cultured with neonatal mouse cardiomyocytes under normoxia or hypoxic/reoxygenation (H/R) conditions, and the culture medium was replaced with fresh DMEM for 4 h. Subsequently, both the culture medium samples and mouse plasma samples were collected following the experimental protocol. The cTnI concentrations in the samples was measured following the manufacturer's instructions. The LDH concentration in the samples was measured using a lactate dehydrogenase assay kit according to the manufacturer's instructions. The cTnI and LDH results were normalized to the volume of the culture medium or plasma.

### 2,3,5‐Triphenyl Tetrazolium Chloride (TTC) Staining

To visualize the infarct area in I/R mice, the heart was rapidly removed and frozen, then the position below the LAD ligating line was cut into four slices and photographed the slices were washed by 0.9% saline and stained with 2.0% TTC (G3005, Solarbio, China) in the dark at 37 °C for 20 min. A stereo‐microscope (Zeiss, Jena, Germany) was used to take images of the slices. The infarct area (pale area) was calculated by Image J software to calculate the percentage of infarct area.

### Nanoparticle Tracking Analysis (NTA)

Absolute size distribution of EVs was measured using the nanoparticle tracking analysis technique, based on the principle that the rate of Brownian movement of nanoparticles in solution is related to their size. Extracellular vesicles purified from 500 µL plasma were resuspended in 1 mL of PBS. The particle size and concentration of the extracellular vesicles were tested using a NanoSight NS300 (Marvel, UK).

### Measurement of Superoxide Dismutase (SOD)

Mouse hearts were subjected to 45 min of ischemia followed by reperfusion at different durations (24 h or 7 days). After whole‐animal perfusion with a solution of 0.9% NaCl containing 0.16 mg mL^−1^ sodium heparin, appropriate heart tissue samples were collected. For sample preparation, 100 µL of SOD Sample Preparation Solution was added per 10 mg of tissue, and the tissues were homogenized at 4 °C or on ice. The homogenates were then centrifuged at 12 000 × g for 3–5 min at 4 °C, and the supernatant was collected for subsequent assays. The activity of SOD was measured using a Total Superoxide Dismutase Assay Kit with WST‐8 (S0101M, Beyotime) according to the manufacturer's instructions.

(1) $$\begin{array}{ccc} & & \;\;\;\text{Inhibition percentage}\\ & & \;\;\;\;=[(\mathrm{Ablank}\;\mathrm{control}1-\mathrm{Ablank}\;\mathrm{control}2)-(\mathrm{Asample}-\mathrm{Ablank}\;\mathrm{control}3)]/\\ & & \;\;\;\;(\mathrm{Ablank}\;\mathrm{control}1-\mathrm{Ablank}\;\mathrm{control}2)\times 100\% \end{array}$$

(2) $$\begin{array}{ccc} & & \text{SOD activity in sample (units)}\\ & & \;=\text{SOD activity in the reaction (units)}\\ & & \;=\text{inhibition percentage}/(1-\text{inhibition percentage}) \end{array}$$

### Measurement of Malondialdehyde (MDA)

MDA content was determined by the Lipid Peroxidation MDA Assay Kit (Beyotime S0131) following the manufacturer's instructions. Mouse hearts were subjected to 45 min of ischemia followed by reperfusion at 24 h. Following whole‐animal perfusion with a solution of 0.9% NaCl containing 0.16 mg mL^−1^ sodium heparin, appropriate heart tissue samples were collected and subsequently lysed. The supernatants from the tissue lysates were obtained, to which 600 µL of thiobarbituric acid (TBA) solution was added. The samples were then incubated at 95 °C for 60 min. A reaction mixture of 200 µL was transferred to a 96‐well plate, and absorbance was measured at a wavelength of 532 nm.

### ATP Production Assay

ATP Assay Kit (Ab83355, Abcam, Cambridgeshire, UK) was used to measure cellular ATP contents according to the manufacturer's protocol. 2 × 10^5^ cardiomyocytes were seeded into 24 well plates for 24 h. Cells were transfected with indicated mimcs and plasmids for 48 h. First, ATP standards of 50 µL were prepared to obtain standard curve. Briefly, centrifuged samples, collected supernatants and transferred to new tubes. 100 µL tissues/cells lysates were mixed with 100 µL ATP reaction mix and incubated for 30 min in the dark. Fluorescence intensity was measured using a microplate reader (Infinite 200 Pro, TECAN).

### Determination of Mitochondrial Complex I level

Mitochondrial Complex I content was measured by a Mitochondrial Complex I (NADH‐CoQ Reductase) Activity Assay Kit (E‐BC‐K149‐M, Elabscience). 1 × 10^6^ cardiomyocytes were seeded into 6 well plates for 24 h. Cells were transfected with indicated mimcs and plasmids for 48 h. The 20 µL mitochondria from tissues/cells were incubated in 20 µL negative/inhibitor reagent at 37 °C for 3 min. Afterward, sample mixture solution was mixed with 200 µL of reaction mix in the dark. The absorbance was measured using a microplate reader (Infinite 200 Pro, TECAN) with emission detection in 0 and 3 min at 340 nm.

### Isolation of Plasma Extracellular Vesicles

Plasma obtained from blood samples was centrifuged at 2000 g at 4 °C for 20 min, then centrifuged at 10 000 g at 4 °C for 10 min to remove cells and platelets. The isolation of extracellular vesicles was performed by ultracentrifugation. Briefly, the plasma was filtered (0.22 µm filter) and ultracentrifuged at 100 000 g at 4 °C for 70 min. Pellets were washed twice by PBS at 100 000 g ultracentrifugation, and finally resuspended in PBS for the subsequent studies. BCA assay was employed to quantify the extracellular vesicles's protein concentration.

### Synthesis of PAA/CaP NPs

The method of synthesis was a modification of the previous work.^[^ ^32^ ^]^ Polyacrylic acid (PAA, Mw≈1800) was obtained from Sigma (USA); while Ca(OH)_2_, isopropyl alcohol (IPA), and (NH_4_)_2_HPO_4_ were supplied by Sinopharm Chemical Reagent Beijing Co., Ltd. In brief, 200 µL PAA solution (Sigma, USA) and 12 mg Ca(OH)_2_ were dispersed in 20 mL deionized water (DI water). After complete dissolution, 40 mL IPA was introduced to the flask under magnetic stirring, and 36 mg (NH_4_)_2_HPO_4_ was added to the flask under stirring for 24 h at room temperature. The obtained PAA/CaP NPs were washed with DI water 3 times and re‐dispersed in DI water for further use.

### Physicochemical Characterization

Transmission electron microscope (TEM) images were obtained using a talos F200x G2 transmission electron microscope (FEI, America) at 200 kV accelerating voltage, whose elemental composition was examined by element‐mapping analysis. Scanning electron microscopy (SEM) images was taken with Gemini 300 (ZEISS, Germany). X‐ray photoelectron spectroscopy (XPS) analysis was carried out using a K‐Alpha (Thermo scientific, USA). Fourier transform infrared (FTIR) spectra was recorded on a Nicolet iS20 (Thermo scientific, USA). UV–vis absorption spectroscopy was carried out on a UV‐2550 spectrophotometer (Shimadzu, Japan). Particle size and zeta potential were analyzed by dynamic light scattering (DLS). The fluorescence intensity of PAA/CaP NPs‐Cy5.5 (440304, Melopeg, China) was measured by a RF‐5301 pc fluorescence spectrophotometer (Shimadzu, Japan).

### The GW4869 Loading Process and Determination of the Loading Efficiency

The 1 mg GW4869 (HY‐19363, MCE, China) was dissolved in 1 mL DMSO. The solution was added to PAA/CaP NPs aqueous solution (60.18 µL, 27.75 mg mL^−1^) and mixed 24 h at room temperature in the dark. Then the mixed solution was centrifuged at 8000 r for 8 min to obtain GW4869‐loaded PAA/CaP NPs. The absorbance spectra of original and supernatant GW4869 were analyzed by UV‐vis at a wavelength of 350 nm. The GW4869 loading efficiency (LE) was calculated using the following formula:

(3) $$\begin{array}{ccc} \mathrm{LE}\;\left(\%\right) & = & [m\;\left(\mathrm{original}\;\mathrm{GW}4869\right)\\ & & -m\;\left(\mathrm{GW}4869\;\mathrm{in}\;\mathrm{supernatant}\right)]/m\left(\mathrm{NPs}\right)\times 100\% \end{array}$$

(4) TheloadingefficiencyofGW4869was75.57±1.69%

### Release Behavior of GW4869 from PAA/CaP NPs

The release profiles of GW4869 from PAA/CaP NPs was investigated using the semipermeable dialysis bag diffusion method at pH 7.4 and 5.0 in PBS at 37 °C, respectively. PAA/CaP NPs containing an equal amount of GW4869 was dispersed in PBS (1 mL, pH 7.4 and 5.0), separately and then transferred into pretreated dialysis bags. Subsequently, the dialysis bags were placed in the corresponding PBS solution (5 mL) at 37 °C. At predetermined intervals, the amount of GW4869 released from the GW4869 loaded NPs into the outside dialysis bags was quantified using a UV‐vis spectrophotometer at a wavelength of 350 nm.

### Hydrophilic Fluorescent Dye Cy5.5 Labeling of PAA/CaP NPs and Chemiluminescence Imaging Staining

The 0.25 mg triSulfo‐Cy5.5 amine (441401, Melopeg) and the 1 mg PAA/CaP NPs were dissolved in 1 mL DI water. Then, mixing was done 24 h at room temperature in the dark. The obtained PAA/CaP NPs‐Cy5.5 were washed with DI water to remove unbound dyes, the precipitate was resuspended and centrifuged at 8,000 g for 8 min three times. The PAA/CaP NPs‐Cy5.5 were intravenously injected into the mice. After 6 h, the mice were anesthetized with Avertin (0.2 g kg^−1^ i.p., Sigma–Aldrich, St Louis, USA). The lung tumor targeting of PAA/CaP NPs was measured based on chemiluminescence imaging by using Living Image Software (Perkin Elmer).

### Cell Culture

Mouse Lewis lung carcinoma (LLC), Tissue culture‐1 (TC‐1) and human embryonic kidney (HEK) 293T cell line were cultured in DMEM supplemented with 10% FBS (Biological Industries, Israel). The cells were cultured in an incubator containing 5% CO_2_ at 37 °C.

### Cell Transfection

Lipofectamine TM 3000 transfection reagent (6 µL) (L3000‐015, Invitrogen, USA) and mimics (50 nM) were respectively diluted in 125 µL Opti‐MEM medium and incubated for 2 min (31985‐070, Gibco, USA). The two diluted regents were added to the cells after incubation for 10 min. Subsequent experiments were carried out 24 h after transfection. The PGC1‐α carrying plasmid was obtained from Genechem (China). The miR‐485‐3p mimics was obtained from GenePharma (China).

The sequences of the constructs used in this study were

miR‐485‐3p mimic sense: 5′‐AGUCAUACACGGCUCUCCUCUC‐3′ and

miR‐485‐3p mimic antisense: 5′‐GAGAGGAGAGCCGUGUAUGACU‐3′;

NC mimics sense: 5′‐UUCUCCGAACGUGUCACGUTT‐3′ and

NC mimics antisense: 5′‐ACGUGACACGUUCGGAGAATT‐3′.

### Cardiomyocytes Co‐Culture with TC‐1 or LLC Cells

For assessing the effects of extracellular vesicles on cardiomyocyte under normoxia or hypoxia/reoxygenation (H/R) model in vitro, co‐culture experiment was performed using cell culture inserts possessing 0.4 mm pores (BD Falcon). Mouse neonatal cardiomyocytes were seeded to the bottom chambers of 6 well‐plates at 5 × 10^5^ cells/well. On the third day, LLC or TC‐1 cells were seeded at the top chambers of the inserts at 2 × 10^5^ cells/insert which gave an almost confluent monolayer. After 24 h, the inserts were removed and the culture media in the bottom chambers were changed with fresh DMEM, the Cardiomyocytes were continued to be cultured under normoxia or hypoxia/reoxygenation (H/R) conditions. Specifically, the normoxia condition is 21% O_2_ 36 h, and the H/R conditions are hypoxia 1% O_2_ 12 h and reoxygenation 21% O_2_ 24 h. Then, the culture media and cardiomyocytes in the bottom chambers were collected and used for the subsequent analysis.

### Cell Counting Kit‐8 (CCK8) Assay

Cell viability was determined by CCK8 assay in 96 well plates. 5 × 10^4^ cardiomyocytes were co‐culture with the relevant plasma extracellular vesicles (20 µg/mL) culturing for 24 h, followed by incubation with 10 µL CCK8 for 70 min. Absorbance was readed at 450 nm using a spectrophotometer (Infinite 200 Pro, TECAN).

### Detection of Reactive Oxygen Species (ROS) Production

5 × 10^5^ cardiomyocytes were plated into 12 well plates and were co‐culture with 1 × 10^5^ TC‐1 or LLC cells for 24 h. ROS of cultured cardiomyocytes and frozen sections of heart tissues were examined by fluorometric ROS Kit (Beyotime, Shanghai, China) according to the manufacturer's instructions. Cultured cells or tissues frozen sections were washed once and incubated with 10 µM DCFH‐DA for 20 min in the dark. Images were acquired by a fluorescence microscope (Olympus).

### Measurement of Mitochondrial Membrane Potential (MMP)

The mitochondrial membrane potential (Δψm) in cardiomyocytes were measured using JC‐1 (Beyotime, Shanghai, China) according to the manufacturer's instructions. 5 × 10^4^ cardiomyocytes were plated into 12 well plates for 24 h. Briefly, the transfected cells were incubated with 10 µM JC‐1 fluorescent dye at 37 °C for 20 min in the dark. Then, the cells were washed and maintained with JC‐1 staining buffer (1 ×). The images of the stained cells were captured by a fluorescence microscope (Olympus).

### Transmission Electron Microscopy

The fresh hearts from experimental mice were harvested, and the ischemic region tissues were cut into 1 mm^3^ pieces and fixed by 2.5% glutaraldehyde. After being washed in 0.1 M sodium cacodylate buffer, tissues were postfixed with 1% buffered osmium. The tissues were dehydrated through the graded alcohol. After the treatment was over to pure acetone and embedding in resin, cells were incubated in a 70 °C oven for 24 h. 70–90 nm ultrathin sections were prepared and stained with uranyl acetate‐lead citrate double staining, subsequently examined by a transmission electron microscope.

### Hematoxylin and Eosin (H&E) Staining

The H&E Staining Kit (G1120, Solarbio, China) was used to demonstrate morphological changes. Paraffin sections of lung tissues were stained with hematoxylin for 5 min and incubated with differentiation solution for 30 s, followed by soaking into water for 15 min. Subsequently, the sections were stained with eosin, followed by dehydration through the graded alcohol and cleaning with xylene. Images were acquired under a microscope (Olympus).

### Masson's Trichrome Staining

After mouse anesthesia, whole hearts were isolated, weighed, fixed in 4% paraformaldehyde (Biosharp, Guangzhou, China), and embedded in paraffin. Mid‐transverse LV sections were cut into 4 µm thick slices using a paraffin sectioning machine (Thermo Fisher Scientific, Waltham, MA, USA). The sections were stained with a Masson's trichrome staining kit (Solarbio, Beijing, China) according to the manufacturer's protocol. The fibrosis fraction was calculated as a percentage of the whole section at 4 × magnification (Olympus), and the fibrotic area was measured using ImageJ software.

### Echocardiographic Measurements

Left ventricular function was analyzed using an echocardiographic system with an ultrasound machine Vevo2100 (Visualsonics, Canada). Mice were anesthetized with 0.2 g kg^−1^ avertin throughout the process for non‐invasive examinations. Medical ultrasound gel (Tianjin Yajie Medical Material Co., Ltd., China) was used as a coupling agent between the ultrasound scan‐head and the skin. The left ventricular parameters including left ventricular end diastolic volume (LVEDV), left ventricular end systolic volume (LVESV), left ventricular internal dimension at end diastole (LVIDd), and left ventricular internal dimension at systole (LVIDs) were measured based on M‐mode recordings. Ejection fraction (EF%) was calculated as EF% = (LVEDV‐LVESV)/LVEDV × 100% and fractional shortening (FS%) as (LVIDd‐LVIDs)/LVIDd × 100%. Statistical analyses were based on the average of measurements of three cardiac cycles.

### Lipophilic Tracer Dil Labeling of Cells and Immunofluorescence Staining

The extracellular vesicles were labeled with 10 µM Dil (C1036, Beyotime, China) for 5 min, and centrifuged at 100 000 g for 70 min to remove unbound dyes, then centrifuged at 100 000 g for 70 min to wash 2 times, finally, the precipitate was resuspended in PBS. The extracellular vesicles were added to myocardial cells and incubated for 12 h. Cells were fixed in 4% formaldehyde and permeabilized with 0.5% Triton‐100 in PBS. Then 5% normal goat serum in PBS was used to block the nonspecific antigen at 37 °C for 1 h. After blocking nonspecific antigen, the cells were washed twice with PBS for 5 min and incubated with primary antibodies against α‐actinin (1:300, 29465, GeneTex, USA) and then incubated with the corresponding second antibodies. Primary antibodies were detected by the fluorescent conjugated secondary antibodies (1:500, anti‐rabbit, A‐11008, Invitrogen). DAPI (Biosharp, Hefei, China) was applied to stain the nucleus. Images were collected with a laser scanning confocal microscope (Zeiss, Jena, Germany).

### In Vivo Uptake of EVs

For tracking of labeled extracellular vesicles, EVs were labeled with DiI. Then, the labeled EVs were washed three times. The labeled EVs (4 × 10^9^ EVs per mouse) were injected into mice via the tail vein. EVs biodistribution were monitored using IVIS Lumina LT series III (PerkinElmer, MA, USA). The mice were euthanized and the hearts ex vivo were examined at 24 h.

### Luciferase Reporter Assay

HEK‐293T cells were transfected with a SV40‐firefly‐Luciferase‐MCS fused with the WT PGC‐1α plasmid (0.1 µg, Genechem, China) and either miR‐485‐3p mimics or a negative control (NC) (50 nM, General Biol, China). Cell lysates were made 48 h after transfection. Renilla and Firefly luciferase activity was measured with the Dual‐Luciferase Kit (Promega, USA) according to the manufacturer's instructions. Firefly normalized to Renilla luciferase ratios were calculated and compared to NC group.

### Small RNA Microarray Analysis

RNA was extracted using the AllPrep DNA/RNA Mini Kit (QIAGEN GmbH, Hilden, Germany). RNA concentration was measured by NanoDrop ND‐1000 and RNA integrity was assessed by standard denaturing agarose gel electrophoresis. Small RNAs profiles were detected using the Arraystar Mouse Small RNA. After sample labeling and array hybridization, raw data was extracted using the Agilent Feature Extraction software (version 11.0.1.1). Quantile normalization, quality control, and differential expression analyses were performed with GeneSpring GX v12.1 software (Agilent Technologies). The Small RNAs showing significant differential expression between the two groups were identified with cutoffs of P < 0.05 and fold change > 1.4. The Small RNA Microarray data generated has been deposited in the Gene Expression Omnibus (GEO) database under accesion code GSE267761 (https://www.ncbi.nlm.nih.gov/geo/query/acc.cgi?acc = GSE267761).

### Quantitative Real‐Time Polymerase Chain Reaction (qRT‐PCR)

Total RNA was extracted with TRIzol reagent (Life Technologies). Nano Drop2000 (Thermo Scientific, Carlsbad, USA) was utilized to verify the quality of RNA samples. Afterward, total RNA (200 ng) was reverse‐transcribed using High‐Capacity cDNA Reverse Transcription Kit (4368813, Thermo Fisher Scientific, USA). QRT‐PCR assay was performed with 1 µL cDNA, 2 µL primers mix, and SYBR Green PCR Master (4913914001, Roche, Switzerland) by a 7500 Fast Real‐Time instrument (Applied Biosystems, USA). The primers of miR‐34b‐3p, pre‐miR‐21c, pre‐miR‐874, and pre‐miR‐324 were obtained from Guangzhou RiboBio Co., Ltd. Other primers were constructed by GenePharma (China). The mRNA expression level was normalized to GAPDH gene, and miRNA level was normalized to U6 gene. The relative quantitative expression was determined using the 2^−ΔΔCT^ method. The primer pair sequences used in the present study are listed in Table S4 (Supporting Information).

### Western Blot

The total proteins were extracted from cells or tissues using RIPA lysis buffer (1 ×) supplemented with a protease inhibitor cocktail (Roche). The concentrations of extracted total protein samples were measured by a BCA Protein Assay Kit (Beyotime). Equal amounts of proteins were separated by SDS‐PAGE and transferred to nitrocellulose filter membranes. After blocking the membranes with non‐fat milk (5% w/v) for 1 h, the proteins were incubated at 4 °C overnight with the primary antibodies against PGC1‐α (1:1000, ab313559, Abcam, UK), Caveolin‐1 (1:1000, 16447‐1‐AP, Proteintech, China), CLTC (1:2000, 26523‐1‐AP, Proteintech), nSMase (1:800, 15239‐1‐AP, Proteintech), GAPDH (1:5000, AC002, Abclonal, USA), or β‐Tubulin (1:5000, AC021, Abclonal). Next, the membranes were incubated with secondary anti‐mouse or anti‐rabbit antibodies (RS23910 and RS23920, ImmunoWay, USA) in dark at room temperature for 1 h. The membranes were scanned, and the gray value of protein band were detected by Odessey CLx (LI‐COR, USA).

### Statistical Analysis

For the animal study, the mice were assigned to randomized groups before experimentation. Experimental analysis was performed in a double‐blind mode. All experimental results were repeated at least three times and are expressed as means ± SEM. Statistical analyses were performed using GraphPad Prism 8.0. The data were tested for normality before parametric statistics was applied using the Shapiro‐Wilk normality test. The equality of variances was tested using the Brown‐Forsythe test. For comparisons between 2 groups, a 2‐tailed Student's test was applied for normal data, and the Mann‐Whitney test was applied for nonnormal data. For comparisons between multiple groups, a 1‐way or 2‐way ANOVA was performed, followed by Tukey's post‐hoc correction for multigroup comparisons. P < 0.05 was considered statistically significant: ^*^ *p* < 0.05; ^**^ *p* < 0.01; ^***^ *p* < 0.001.

## Conflict of Interest

The authors declare no conflict of interest.

## Author Contributions

Z. M., Q. L., G. L. and M. Z. contributed equally to this work. Z.T.M., Q.L., G.X.L., M.J.Z., J.X.F., X.T.H., X.Q.H., Z.W.Q., R.H.L., C.L., H.Y.J., Y.C.D., Y.Y., B.F.Y., and W.J.D. performed research; Z.T.M., Q.L., G.X.L. analyzed data; Y.Y., Z.T.M., B.F.Y., W.J.D. designed the study and wrote the manuscript. All authors have read and approved the article.

## Supporting information



Supporting Information

## Data Availability

All data were available from the corresponding author upon reasonable request.
