# Supplementary material for: Tumor Extracellular Vesicles Aggravate Mitochondrial Damage in Myocardial Ischemia/Reperfusion Injury
Source: Adv Sci (Weinh). 2025 Aug 11;12(35):e17493. doi: 10.1002/advs.202417493 (PMC12462958; doi:10.1002/advs.202417493)
Supplement: Supplementary file 1 — Supporting Information [file ADVS-12-e17493-s001.docx]

Supplementary Materials for

**Tumor Extracellular Vesicles** **Aggravate Mitochondrial Damage in Myocardial Ischemia/Reperfusion Injury**

Zhongting Mei, Qian Liu, Guoxin Liu, Manjie Zhang, Jiaxin Fang, Xuting He, Xueqi He, Zhengwei Qin, Ronghua Liu, Chuang Liu, Hongyu Ji, Yuechao Dong, Ye Yuan, Baofeng Yang, Weijie Du

**Correspondence to:** Prof. Weijie Du [duweijie@hrbmu.edu.cn](mailto:duweijie@ems.hrbmu.edu.cn); Prof. Baofeng Yang yangbf@ems.hrbmu.edu.cn; Prof. Ye Yuan [yuanye_hmu@126.com](mailto:yuanye_hmu@126.com)

This file includes:

Supplementary Figures S1-S14

Supplementary Table S1-S4


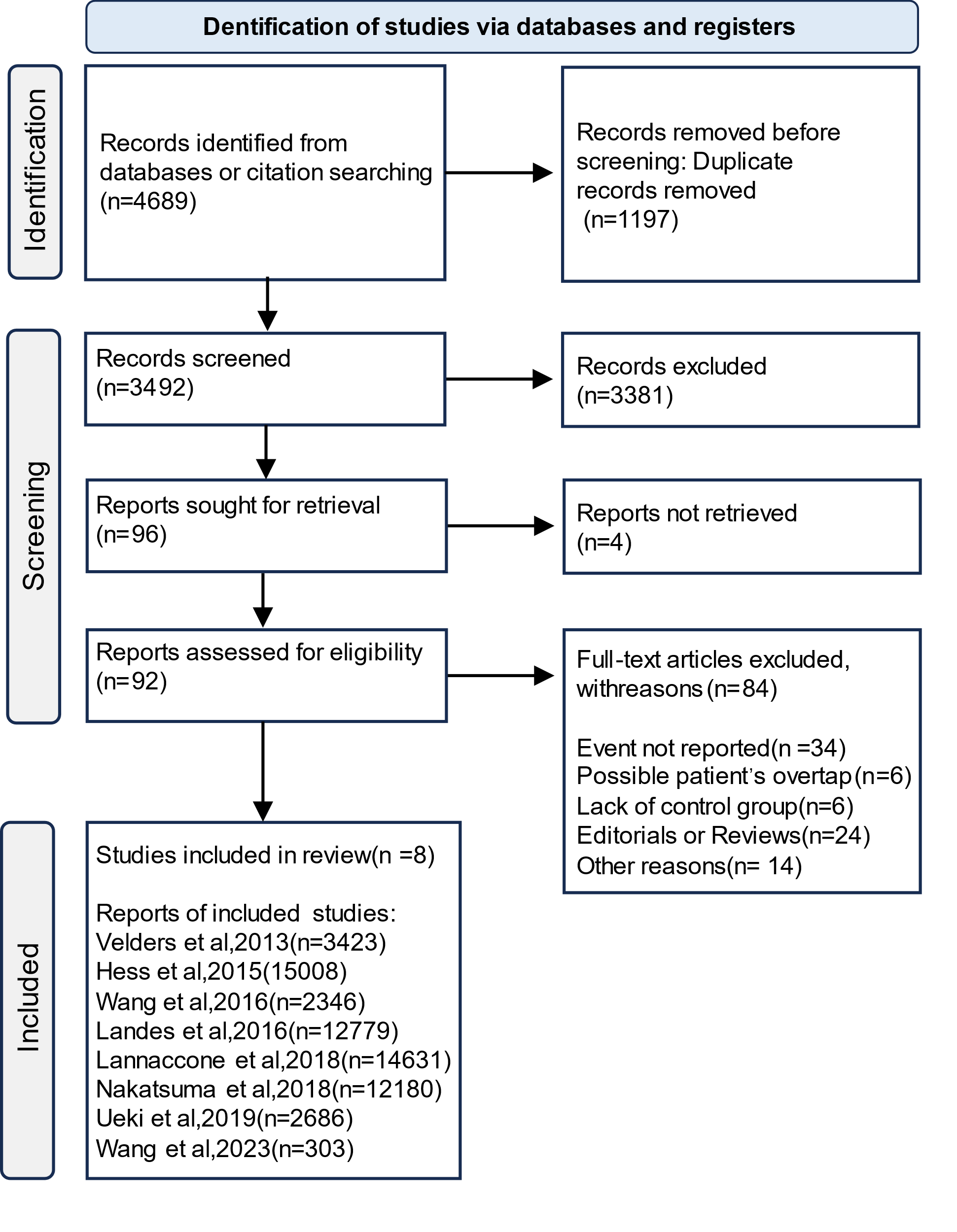


**Supplementary Figure S1.** PRISMA flow diagram showing the selection process of studies.

**
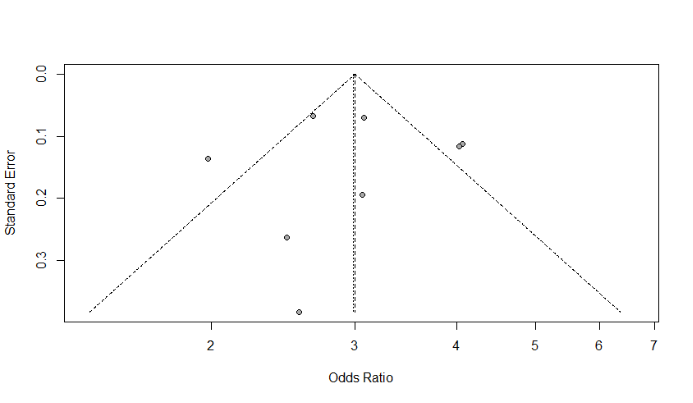
**

**CV death**

**
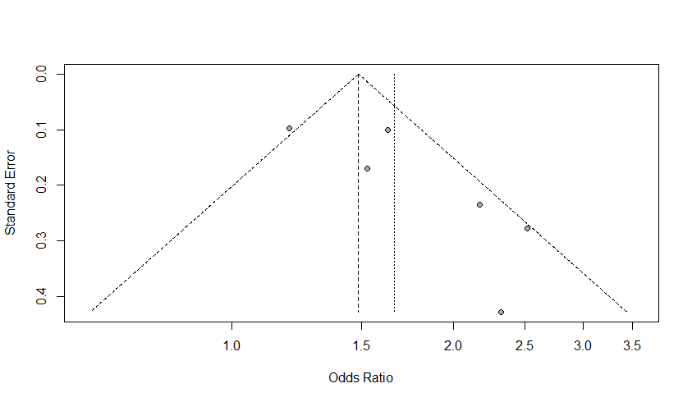
Supplementary Figure S2.** Funnel plots for the assessed outcomes.

**All-cause death**

**
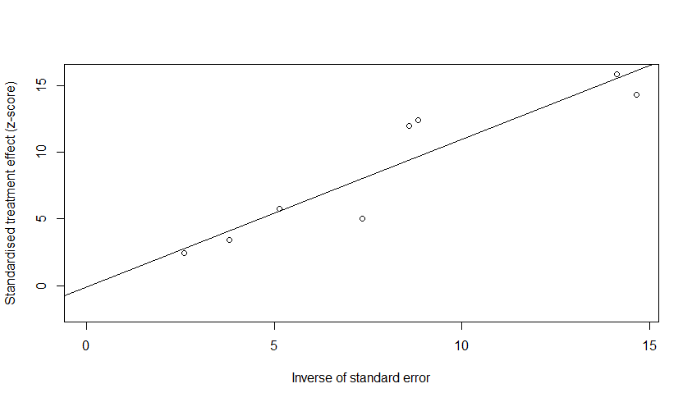
**

**CV death**

P=0.947

**All-cause death**

**
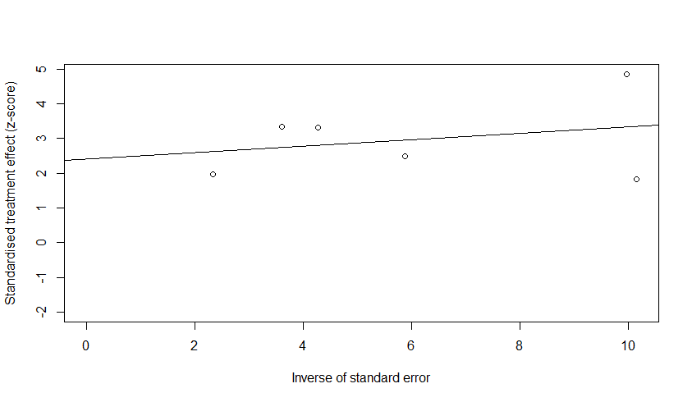
Supplementary Figure S3.** Egger’s publication bias plots for the assessed outcomes.

P=0.094


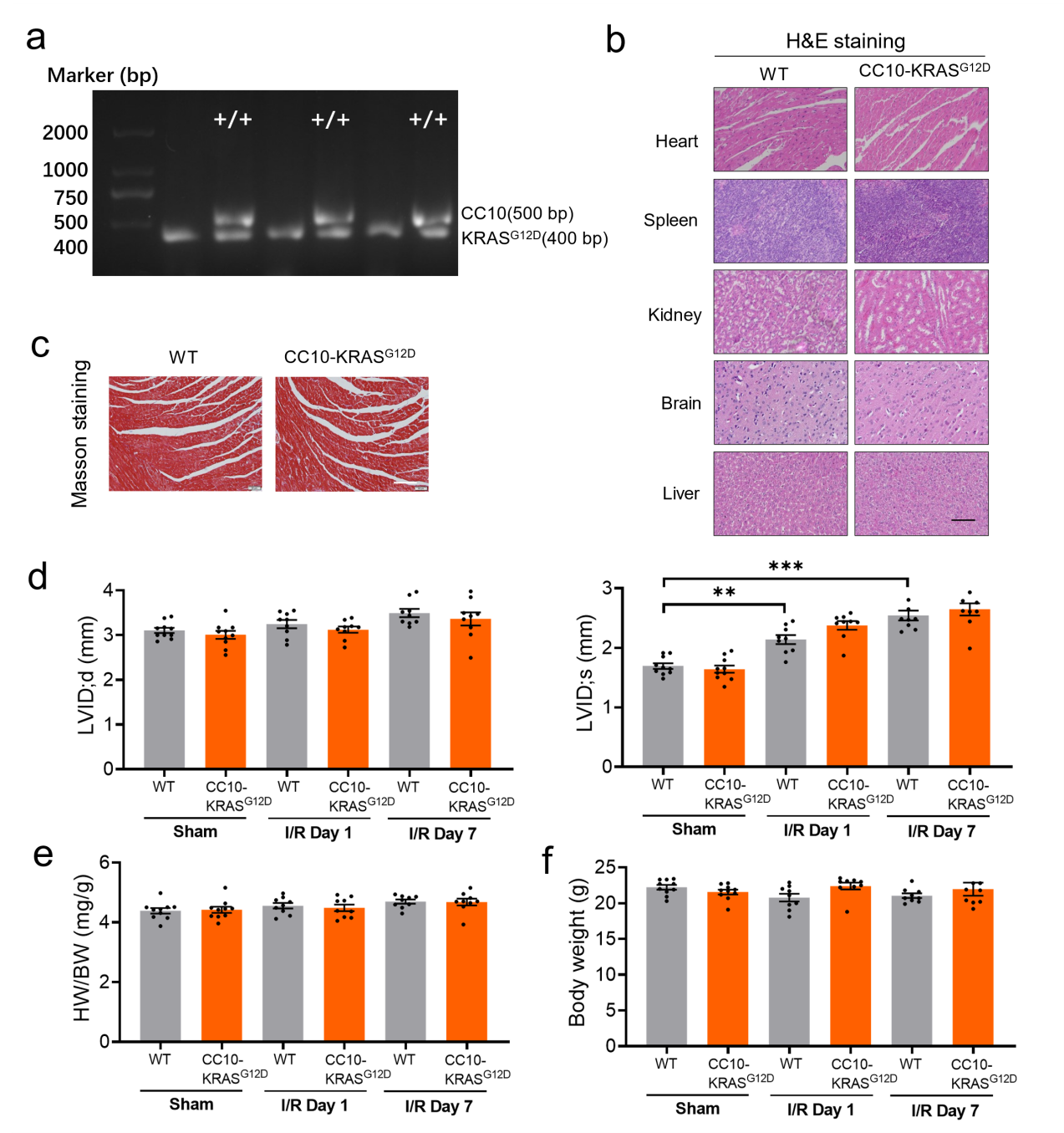


**Supplementary Figure S4.** a) Genotyping of offspring of CC10-KRAS^G12D^ (+/+) female and male mice treated with the doxycycline diet, CC10 (500 bp), KRAS^G12D^ (400 bp), (+/+) is double positive; b) Representative images of H&E staining of heart, spleen, kidney, brain, and liver in WT and CC10-KRAS^G12D^ mice (Bar: 100 μm); c) Representative images of Masson's trichrome-stained transverse mid slices of LVs in WT and CC10-KRAS^G12D^ (Bar: 100 µm); d) Statistics of left ventricular internal dimension at systole (LVIDs) and left ventricular internal dimension at end-diastole (LVIDd) (N=9-10/group); e-f) Heart weight to body weight ratio and body weights in WT and CC10-KRAS^G12D^ mice with sham/IR operated conditions (N=9-10/group).


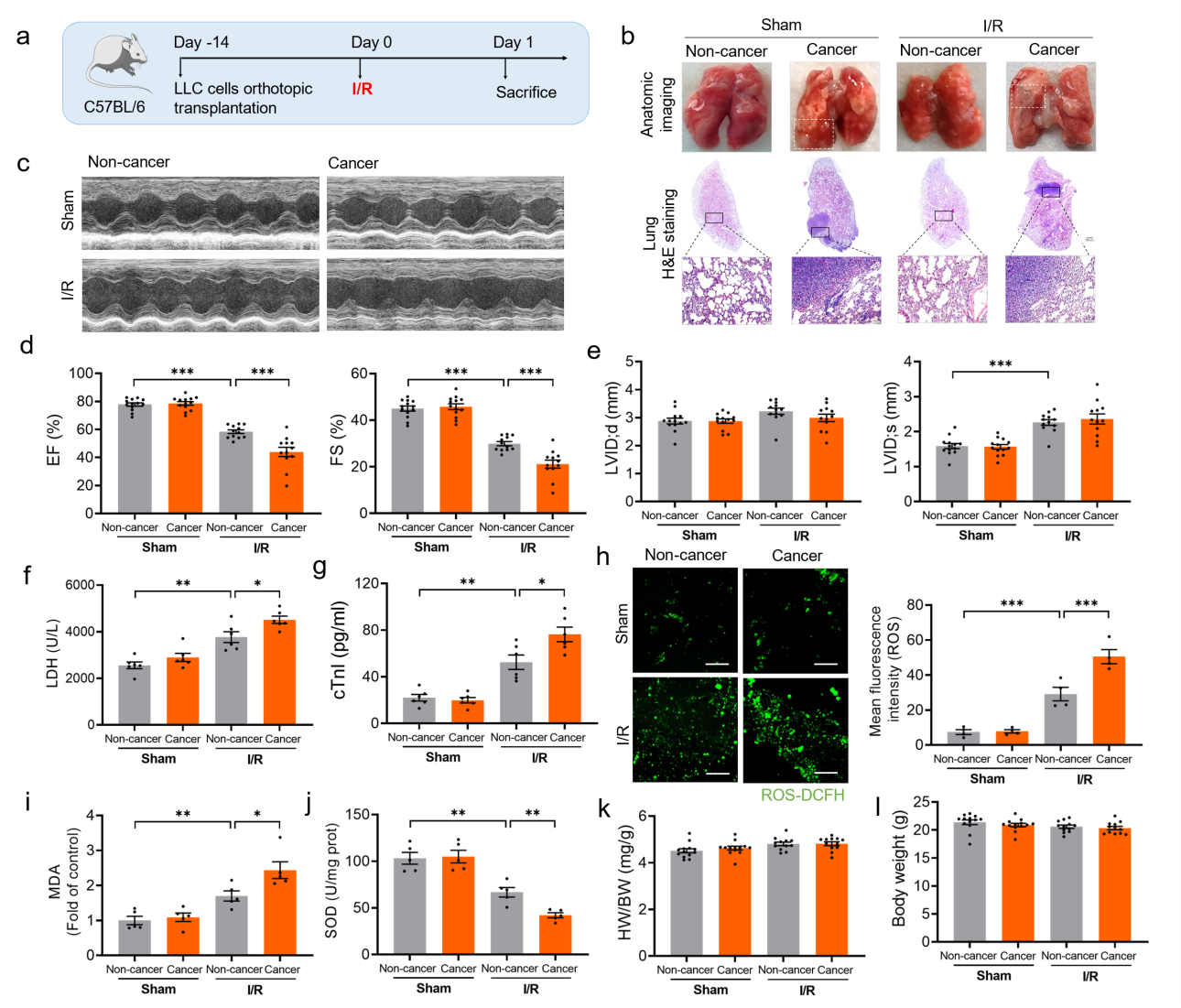


**Supplementary Figure S5.** a) Schematic presentation on the time-line of in vivo animal model construction experiment; b) Representative images of anatomic imaging and H&E staining of lung tumors in mice; c-e) Representative images of echocardiographs and statistical data on EF%, FS%, LVIDd, LVIDs (N=12-13/group); f-g) Plasma LDH and cTnI levels were detected in mice (N=6/group); h) Representative images and quantitative results of lipid-ROS using ROS fluorescence staining (Bar: 40 μm) (N=4/group); i-j) The levels of MDA and SOD were measured by ELISA assays (N=5/group); k-l) Heart weight to body weight ratio and body weights in non-cancer and cancer mice with sham/IR operated conditions (N=9-10/group).


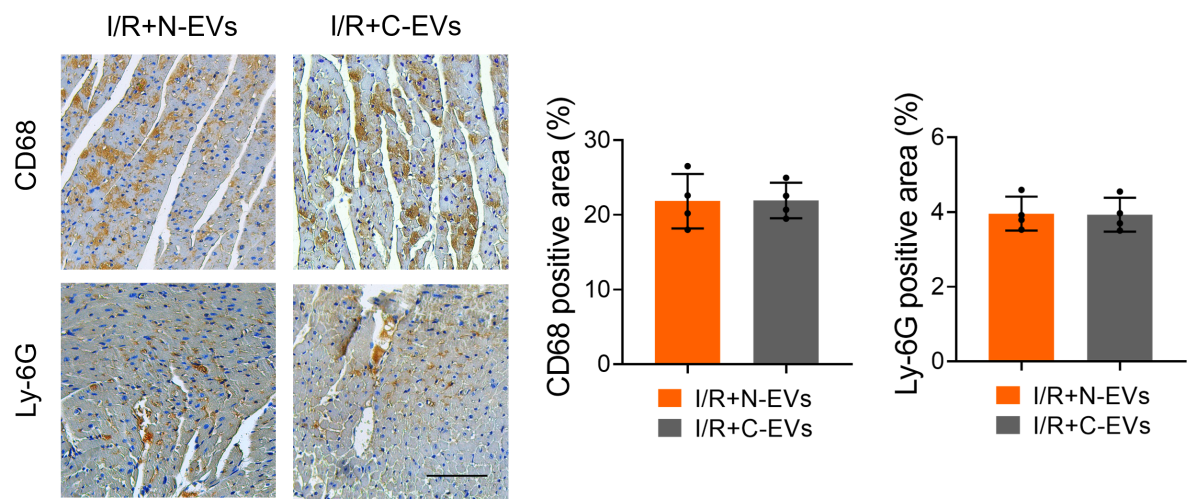


**Supplementary Figure S6.** Quantification and representative immunohistochemical images of CD68, Ly-6G staining in EVs-treated mice heart tissues (N=4/group) (Bar: 100 μm).


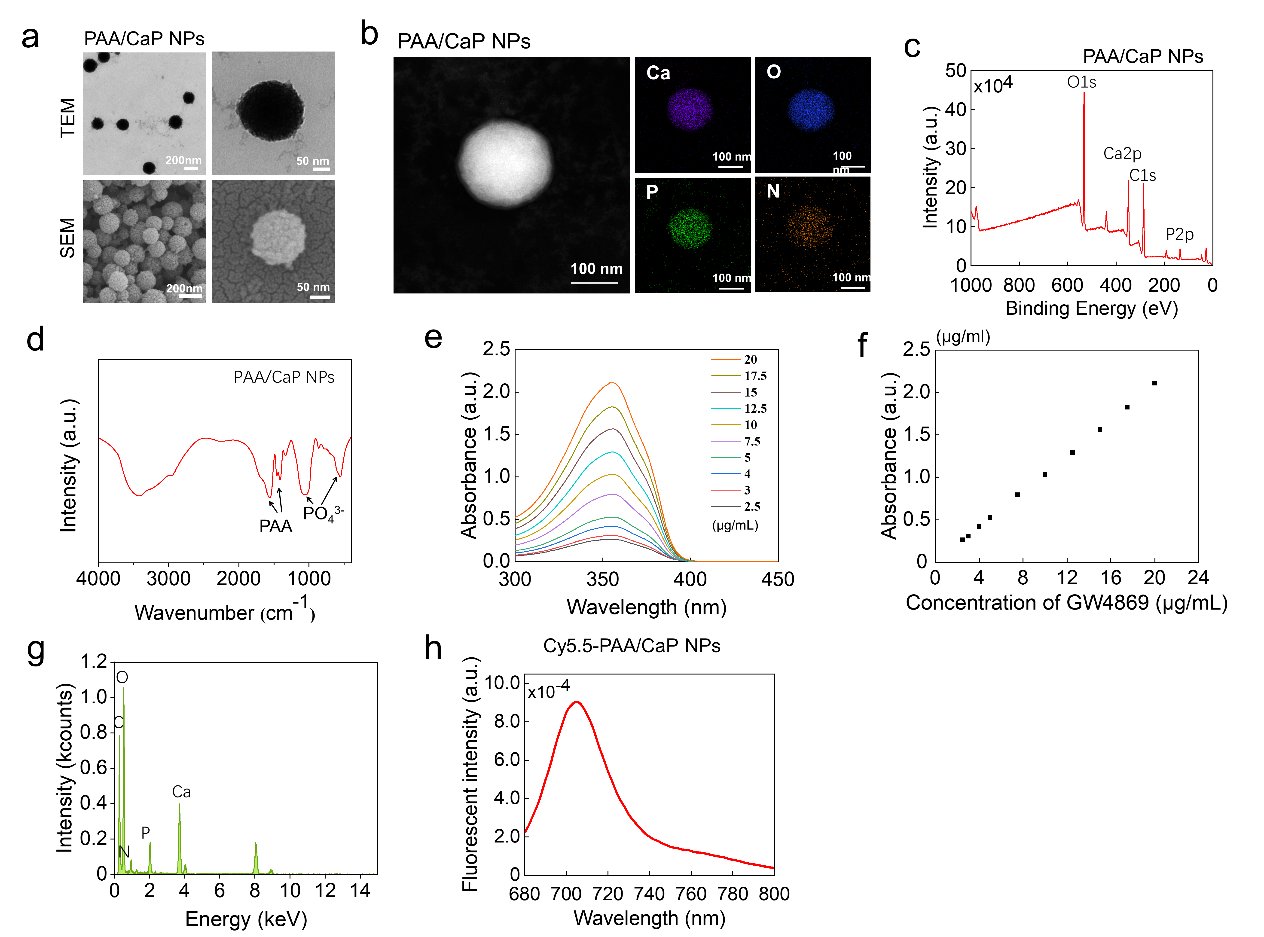


**Supplementary Figure S7.** a) TEM and SEM images of PAA/CaP NPs; b) HRTEM image and the elemental mapping images of individual PAA/CaP NPs; c) XPS spectrum of PAA/CaP NPs; d) FTIR spectra of PAA/CaP NPs; e) The UV-vis absorption spectra of GW4869 at a series of concentrations; f) Standard curve of GW4869 using UV spectrometry at 350 nm. Y=0.1048X-0.0018, R^2^=0.9998 (Y: UV value, X: concentration (μg/ml); g) EDX spectrum of PAA/CaP NPs; h) The fluorescence intensity of PAA/CaP NPs-cy5.5.


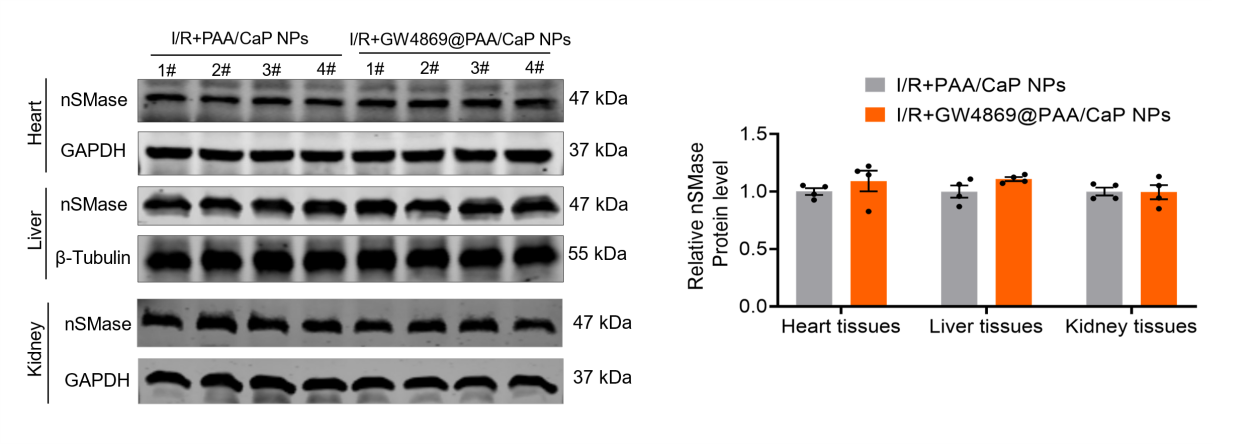


**Supplementary Figure S8.** Western blot detected the protein expression level of nSMase in heart, liver, and kidney tissues (N=4/group).


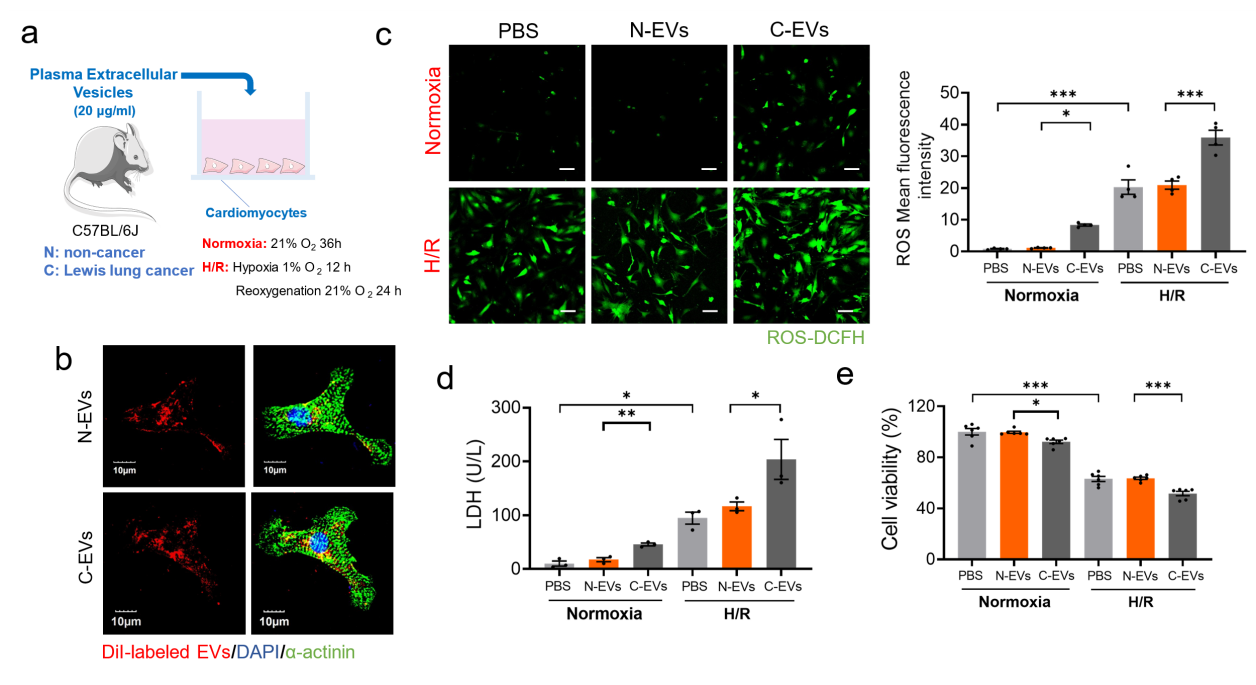


**Supplementary Figure S9.** a) The schematic presentation of cardiomyocytes cultured with plasma extracellular vesicles (20 μg/mL) from C57BL/6J mice; b) Representative images of immunofluorescence co-staining showing the uptake of N-EVs and C-EVs by cardiomyocytes after co-culturing with Dil-labelled extracellular vesicles for 12 h (Bar: 10 μm). Extracellular vesicles were labelled with Dil (red), nuclei were labelled with DAPI (blue), and cardiomyocytes were labelled with α-actinin (green); c) Representative images and quantitative results of lipid-ROS using ROS fluorescence staining (N=4/group, Bar: 40 μm); d) The Cardiomyocyte culture medium LDH was measured by ELISA assay (N=3/group); e) Cardiomyocyte viability was detected by CCK-8 assay (N=6/group).


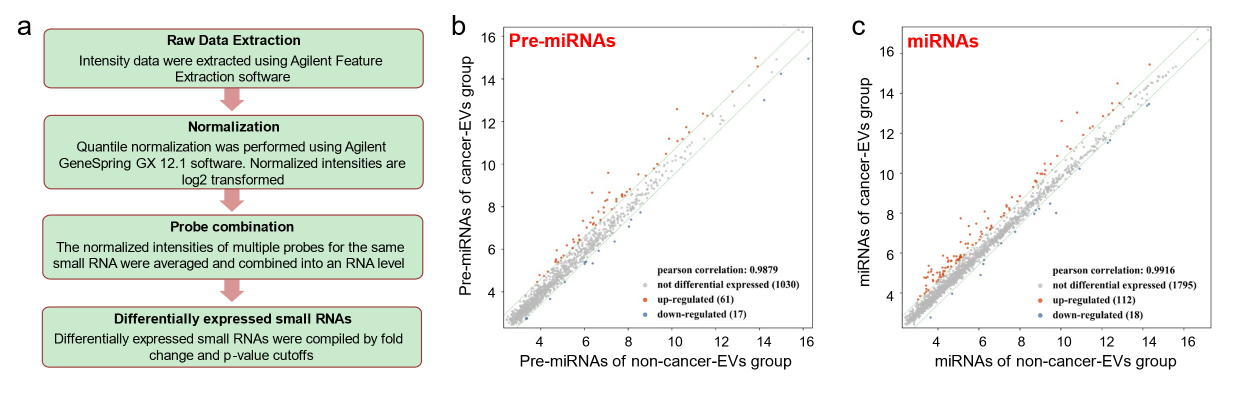


**Supplementary Figure S10.** a) Analysis flowchart for the Small RNA microarray data; b) The volcano plot below was generated based on all expressed pre-miRNAs, the red-blue gradient color points represent significantly up-regulated and down-regulated pre-miRNAs between non-cancer-EVs and cancer-EVs group; c) The volcano plot below was generated based on all expressed miRNAs, the red-blue gradient color points represent significantly up-regulated and down-regulated miRNAs between non-cancer-EVs and cancer-EVs group.


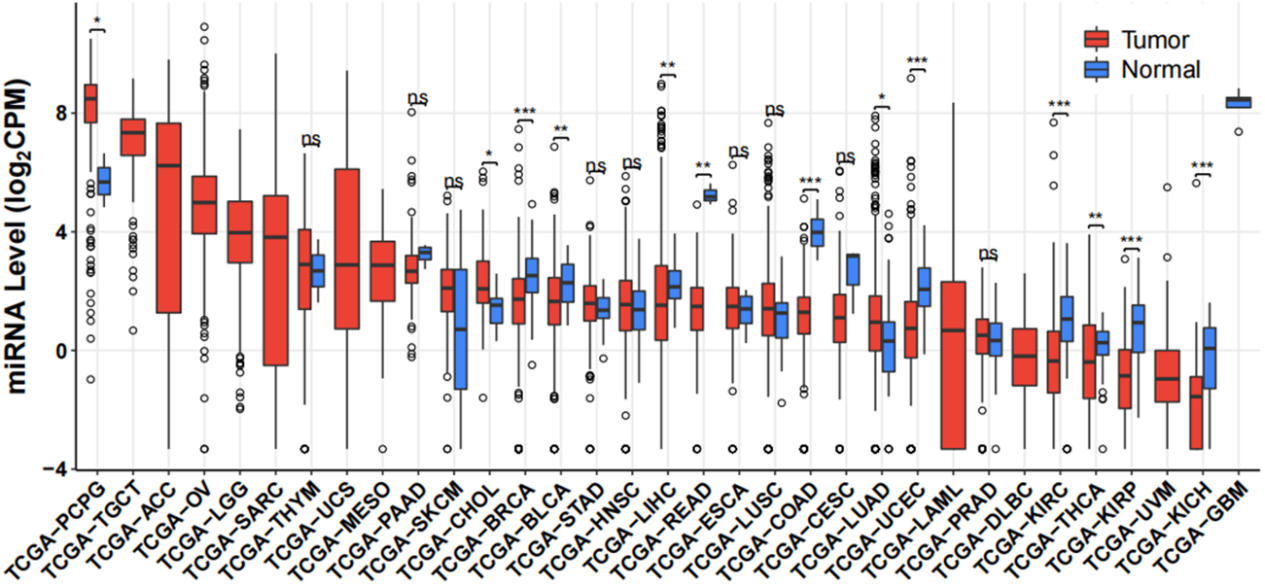


**Supplementary Figure S11.** The expression of miR-485-3p in tumor and normal samples from the Cancer Genome Atlas (TCGA) miRNome database.

#
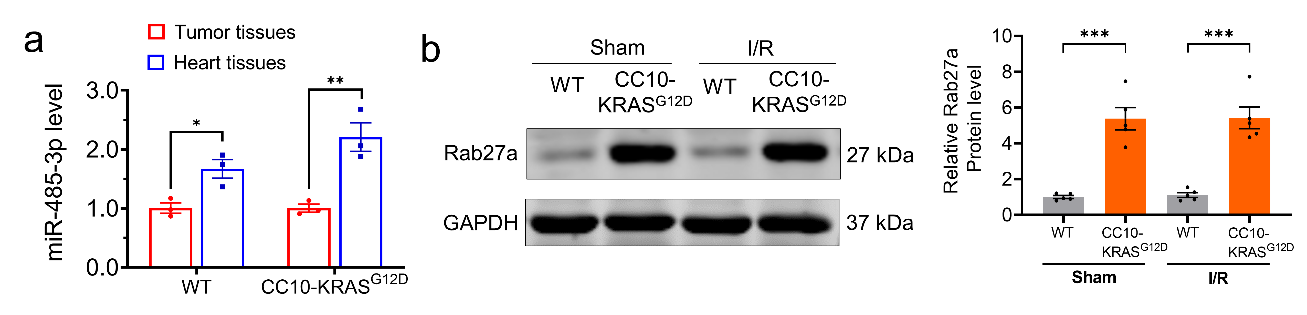


**Supplementary Figure S12.** a) The expression of miR-485-3p with tumor and heart tissues from WT and CC10-KRAS^G12D^ mice (N=3/group); b) The protein expression levels of Rab27a were determined by western blot in lung and tumor tissues (N=5/group).


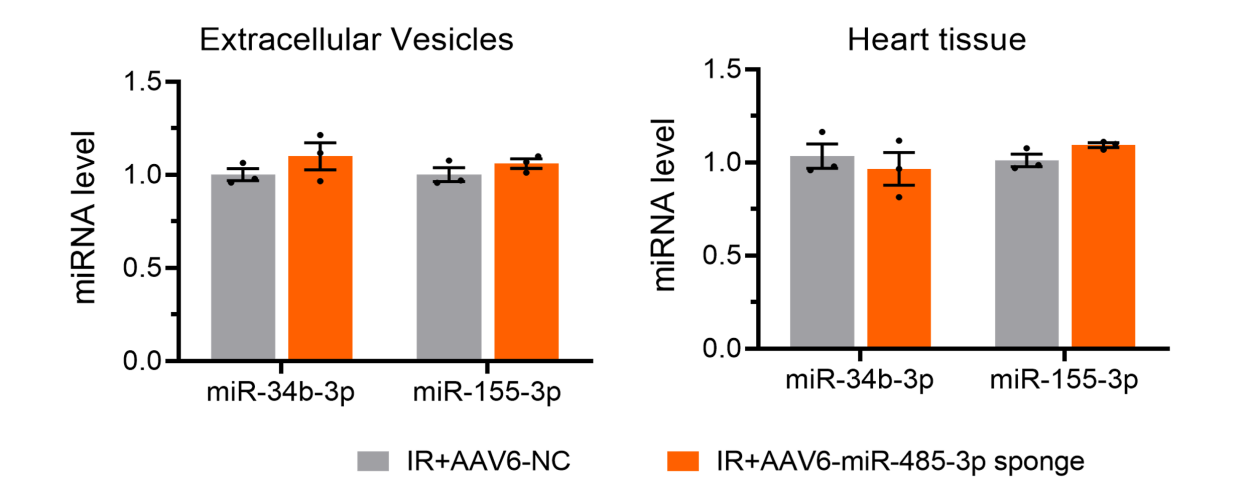
.

**Supplementary Figure S13.** qRT-PCR analysis on the expression levels of miR-34b-3p and miR-155-3p in plasma extracellular vesicles and heart tissues from CC10-KRAS^G12D^ mice treated with AAV6-miR-485-3p sponge/NC (N=3/group).

#
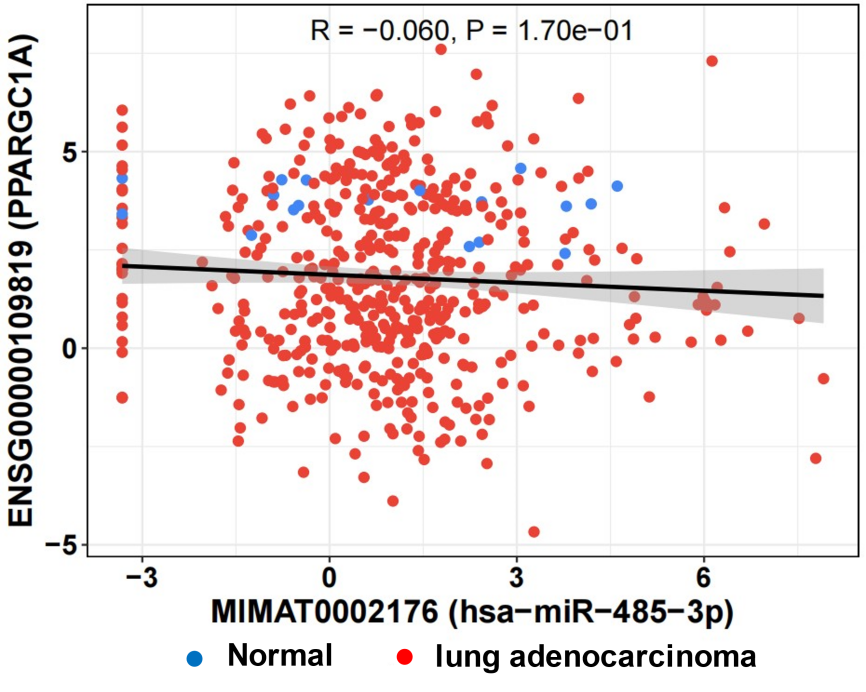


**Supplementary Figure S14.** The correlation of miR-485-3p and PPARGC1A/PGC-1α in lung adenocarcinoma and normal samples from the Cancer Genome Atlas (TCGA) miRNome database.

**Supplementary Table S1. PICO criteria for the systematic review and meta-analysis**

| **PICO elements** |  | **Search terms** | **Search strategies** |
| --- | --- | --- | --- |
| **P**atient or **P**opulation | Patients undergoing PCI | Cancer | Cancer |
| **I**ntervention | Patients with cancer | PCI | Percutaneous coronary intervention OR PCI |
| **C**omparison | Patients without cancer | - | - |
| **O**utcome | Mortality | - | - |

PCI, percutaneous coronary intervention.

**Supplementary Table S2. Baseline clinical characteristics of the patients in the studies included in the meta-analysis**

| **Study** | **Source** | **Design** | **N. of pts** | **Follow-up (years)** | **Age** | **Male (%)** | **HTN (%)** | **DM (%)** | **Dys-lipidemia (%)** | **Smoking (%)** | **Prior MI (%)** | **ACS (%)** | **DES (%)** |
| --- | --- | --- | --- | --- | --- | --- | --- | --- | --- | --- | --- | --- | --- |
| Velders et al  2013 | Dutch registry | Prospective | 3,423 | 1 | 63.2 | 75 | 35.8 | 11.3 | 23 | 45.8 | 10.8 | NA | 70.2 |
| Hess et al  2015 | DISSC at Duke University | Retrospective, registry | 15,008 | 7.6 | 62.2 | 66.3 | 65.4 | 27.6 | NA | 53 | 51.1 | 72.2 | 61.9 |
| Wang et al  2016 | Mayo Clinic Cath Lab PCI Registry database | Retrospective, registry | 2346 | 10 | 64.3 | 70.7 | 69.4 | 18.1 | NA | 67.9 | 14.7 | NA | 53.2 |
| Landes et al  2017 | Rabin insitution database | Retrospective | 12,779 | 6.4 | 76.9 | 72.2 | 87 | 45.7 | NA | 21.2 | NA | 57.5 | 47.4 |
| Iannaccone et al  2018 | BleeMACS registry | Retrospective, registry | 14,631 | 1 | 63.3 | 76.9 | 58.2 | 23.8 | 52.9 | NA | NA | 11.7 | 39.8 |
| Nakatsuma et al  2018 | CREDO-Kyoto registry Cohort-2 | Retrospective, registry | 12,180 | 5.3 | 68.3 | 72.2 | 82 | 38.2 | NA | 31.9 | 10.1 | 27.2 | 55.5 |
| Ueki et al  2019 | Bern University Hospital | Retrospective, registry | 2686 | 1 | 67.7 | 74 | 69.2 | 23 | NA | 26.9 | 17 | NA | NA |
| Wang et al  2023 | The 2nd Affiliated Hospital of Harbin Medical University | Retrospective | 303 | 1 | 65 | 64.7 | 50.3 | 37.9 | 37.9 | 30.1 | 5.9 | NA | NA |

ACS, acute coronary syndrome; DES, drug-eluting stent; DM, diabetes mellitus; HTN, hypertension; MI, myocardial infarction; NA, not available; pts, patients.

**Supplementary Table S3. Quality of the studies included in the meta-analysis**

| **Author** | **Selection** | | | | **Comparability of cohorts** | **Outcome** | | | **Total score** |
| --- | --- | --- | --- | --- | --- | --- | --- | --- | --- |
|  | **Exposed cohort represent-tativeness** | **Non-exposed cohort selection** | **Exposure ascertain-ment** | **Absence of outcome at baseline** |  | **Outcome ascertain-ment** | **Length of follow-up** | **Follow-up adequacy** |  |
| Velders et al  2013 | * | * | * | * | ** | * | * | * | 9 |
| Hess et al  2015 | * | * | * | * | ** | * | * | * | 9 |
| Landes et al  2017 | * | * | * | * | * | * | * | * | 8 |
| Iannaccone et al  2018 | * |  | * | * | * | * | * | * | 7 |
| Nakatsuma et al  2018 | * | * | * | * | * | * | * | * | 8 |
| Wang et al  2016 | * | * | * | * | ** | * | * | * | 9 |
| Ueki et al  2019 | * | * | * | * | * | * | * | * | 8 |
| Wang et al  20 | * | * | * | * | ** | * | * | * | 9 |

The Newcastle-Ottawa scale uses a star system (0 to 9) to evaluate the selected studies in three domains: selection, comparability, and outcomes. Star (*) indicates that the item is present. For each item, the maximum score is 1 star (*) for selection and outcome components and 2 stars (**) for comparability components. Higher scores represent higher study quality.

**Supplementary Table S4. Primer sequences for qRT-PCR**

| **Primers** | **Primer sequences (5`-3`)** |
| --- | --- |
| mmu-GAPDH | F: ACCACAGTCCATGCCATCAC  R: TCCACCACCCTGTTGCTGTA |
| U6 | F: GCTTCGGCAGCACATATACTAAAAT  R: CGCTTCACGAATTTGCGTGTCAT |
| mmu-miR-485-3p | RT: GTCGTATCCAGTGCAGGGTCCGAGGTATTCGCACTGGATACGACGAGAGG  F: AGTCATACACGGCTCTCCTCTC  R: ATCCAGTGCAGGGTCCGAGG |
| mmu-miR-155-3p | RT: GTCGTATCCAGTGCAGGGTCCGAGGTATTCGCACTGGATACGACGTTAAT  F: CCCCTCCTACCTGTTAGCATTAAC  R: ATCCAGTGCAGGGTCCGAGG |
| mmu-Ndufa1 | F: CCGGAAGAGAGGTAAAGCCG  R: ACATCTCCGCACCGTTACTC |
| mmu-Ndufa3 | F: GGCCACACCCTACAACTACC  R: CAGGCATGTTCCCGTCATCT |
| mmu-Ndufa5 | F: CCACACGAGAGGCTCACAAT  R: TAACATCTGGCTCCGCCTTG |
| mmu-Ndufa11 | F: CCTGTTCAAGATCGGCAAGC  R: CCATCTCATGCAGCAAGCCT |
| mmu-Ndufb8 | F: GGAATCGTGTGGACACGTCA  R: GTACTGCTTCGGACCCACAG |
| mmu-Ndufb10 | F: CAGCATGCCAAGAACCGAAC  R: TGTGATGTCTGGCACTCGAC |
| mmu-Ndufb11 | F: CCAGAACCCGAGGACGAAAA  R: ATTCCAGACGTCCACCACAG |
| mmu-Ndufs1 | F: AAGCCATCGCTCGTCTCATT  R: TCTCCCTTCACAAATCGGGC |
| mmu-Ndufs2 | F: TGCACCAGATGCTGACCAAC  R: CCACCTGGTCGTAAACGTCA |
| mmu-Ndufs4 | F: ACAGTTCCCTGCACAGACAG  R: AGCTGATTCCAGGCACTCAC |
| mmu-Ndufv1 | F: AAGCCATCGCTCGTCTCATT  R: TCTCCCTTCACAAATCGGGC |
